# Niche Laminin and IGF-1 Additively Coordinate the Maintenance of Oct-4 Through CD49f/IGF-1R-Hif-2α Feedforward Loop in Mouse Germline Stem Cells

**DOI:** 10.3389/fcell.2021.646644

**Published:** 2021-07-26

**Authors:** Heng-Kien Au, Syue-Wei Peng, Chin-Lin Guo, Chien-Chia Lin, Yi-Lin Wang, Yung-Che Kuo, Tsz-Yau Law, Hong-Nerng Ho, Thai-Yen Ling, Yen-Hua Huang

**Affiliations:** ^1^Taipei Medical University (TMU) Research Center of Cell Therapy and Regeneration Medicine, Taipei Medical University, Taipei, Taiwan; ^2^Department of Obstetrics and Gynecology, School of Medicine, College of Medicine, Taipei Medical University, Taipei, Taiwan; ^3^Department of Obstetrics and Gynecology, Taipei Medical University Hospital, Taipei, Taiwan; ^4^Center for Reproductive Medicine, Taipei Medical University Hospital, Taipei Medical University, Taipei, Taiwan; ^5^International Ph.D. Program for Cell Therapy and Regeneration Medicine, College of Medicine, Taipei Medical University, Taipei, Taiwan; ^6^Department of Biochemistry and Molecular Cell Biology, School of Medicine, College of Medicine, Taipei Medical University, Taipei, Taiwan; ^7^Graduate Institute of Medical Sciences, College of Medicine, Taipei Medical University, Taipei, Taiwan; ^8^Institute of Physics, Academia Sinica, Taipei, Taiwan; ^9^Department of Obstetrics and Gynecology, Taipei Municipal Wanfang Hospital, Taipei, Taiwan; ^10^Department and Graduate Institute of Pharmacology, College of Medicine, National Taiwan University, Taipei, Taiwan; ^11^Comprehensive Cancer Center of Taipei Medical University, Taipei, Taiwan; ^12^The Ph.D. Program for Translational Medicine, College of Medical Science and Technology, Taipei Medical University, Taipei, Taiwan

**Keywords:** germline stem cell, niche, extracellular matrix, hypoxia, laminin, IGF, self-renewal

## Abstract

The mechanism on how extracellular matrix (ECM) cooperates with niche growth factors and oxygen tension to regulate the self-renewal of embryonic germline stem cells (GSCs) still remains unclear. Lacking of an appropriate *in vitro* cell model dramatically hinders the progress. Herein, using a serum-free culture system, we demonstrated that ECM laminin cooperated with hypoxia and insulin-like growth factor 1 receptor (IGF-1R) to additively maintain AP activity and Oct-4 expression of AP^+^GSCs. We found the laminin receptor CD49f expression in d2 testicular GSCs that were surrounded by laminin. Laminin and hypoxia significantly increased the GSC stemness-related genes, including Hif-2α, Oct-4, IGF-1R, and CD49f. Cotreatment of IGF-1 and laminin additively increased the expression of IGF-IR, CD49f, Hif-2α, and Oct-4. Conversely, silencing IGF-1R and/or CD49f decreased the expression of Hif-2α and Oct-4. The underlying mechanism involved CD49f/IGF1R-(PI3K/AKT)-Hif-2α signaling loop, which in turn maintains Oct-4 expression, symmetric self-renewal, and cell migration. These findings reveal the additive niche laminin/IGF-IR network during early GSC development.

## Introduction

Germline stem cells (GSCs), including primordial germ cells (PGCs), postmigratory PGCs, and spermatogonial stem cells, are cells involving gamete production. During the embryogenesis, germ cells begin with early PGC specification through the expression of transcription factors, such as Fragilis, Stella, and Blimp1 to regulate PGC emergence (Saitou et al., 2002; Ohinata et al., 2005), and then followed by external BMP4 signaling that enables PGC competence (Lawson et al., 1999; Ying et al., 2000, 2001). In response to surrounding niche factors, competent PGCs can then either maintain self-renewal or migrate into the genital ridge, where they become mature germ cells. The robustness of these cellular behaviors requires the synergistic coordination of responses to niche factors, and defects in the self-renewal or maintenance of stemness during early germ cell development cause insufficient germ cell production in embryonic gonads, which can lead to infertility or formation of extragonadal germ cell tumors (Hoei-Hansen et al., 2006). Currently, the understanding of signaling and networking interactions among niche factors is still limited.

Niche factors have been referred to as secreted growth factors, cytokines, and morphogens. Recently, other factors, including oxygen level and extracellular matrix (ECM) composition, have been considered critical for maintaining the stemness of germ cells (Ginsburg et al., 1990; Lawson et al., 1999; Ying et al., 2000; Saitou et al., 2002; Ohinata et al., 2005). For example, a physiological hypoxic environment (with 1–5% O_2_), which stabilizes hypoxia-inducible factor (HIF) by preventing its degradation (Ginouves et al., 2008), was reported to play a crucial role in embryogenesis and early germ cell survival (Scortegagna et al., 2005; Covello et al., 2006). Increasing Hif-2α expression by generating Hif-2α knockin mice was reported to enhance expression of the pluripotent transcription factor Oct-4 through direct promoter binding, whereas the loss of Hif-2α results in a severe deficiency of embryonic PGCs in the genital ridge (Covello et al., 2006) and subsequent azoospermia (Scortegagna et al., 2005).

In parallel, evidence has recently identified the association between ECM components and the regulation of stem cell fate (Mercier, 2016), where the signaling of ECM often acts through the cell surface receptor integrin, which is composed of various α and β subtypes specific to ECM components (Ying et al., 2001). Laminin is the major ECM component involved in stem cell self-renewal, migration, adhesion, and differentiation (Covello et al., 2006). It binds primarily to the heterodimeric integrins α3β1, α6β1, and α6β4 (Lawson et al., 1999). *In vitro* assay has shown that laminin maintains Oct-4/Sox2 expression and cell proliferation in embryonic stem cells (Ying et al., 2000; Ohinata et al., 2005). Overexpression of integrin α6, one of the integrin subunit specific to laminin, was found to enhance the proliferation, differentiation, and Oct-4/Sox2 expression in human mesenchymal stem cells *via* the PI3K/Akt/p53 pathway (Scortegagna et al., 2005).

Hypoxic response and ECM signaling might act along or coordinate with growth factor–mediated signaling to determine the fate of stem cells (Francis and Wei, 2010). Decoupling of the effects of these factors *in vivo* is unlikely because a single genetic deletion often causes embryonic fatality. Moreover, the use of conventional *in vitro* stem cell cultures is not possible because most of cell cultures use a serum-containing medium, which does not permit a distinction to be made between the effects of hypoxia and ECM and those mediated by growth factor signaling. To overcome this limitation, we previously developed a serum-free culture system to generate pluripotent GSCs by using wild-type neonatal mouse testes (Huang et al., 2009). These pluripotent GSCs, referred to as CD49f^+^AP^+^GSCs, display early germ cell characteristics, including high alkaline phosphatase (AP) activity, an abundance of cell surface proteins, such as the stage-specific embryonic antigen (SSEA)-1 and the laminin receptor integrin α6 (also referred to as CD49f), an ability to express PGC-related genes (e.g., *Oct-4*, *Nanog*, and *Blimp1*), and the capability to migrate and differentiate into multiple cell types *in vitro* as well as to form chimeras/teratomas *in vivo* (Huang et al., 2009). Using the CD49f^+^AP^+^GSCs as an *in vivo* model system, we demonstrated that the niche hypoxia-induced growth factor signaling, Hif-2α-IGF-1R, can maintain the expression of Oct-4 and the capacity of self-renewal in embryonic GSCs (Huang et al., 2009, 2014). However, whether laminin also plays a crucial role in maintaining GSC stemness and survival, and whether crosstalk occurs between laminin signaling and the signaling from other niche factors, such as hypoxia and growth factor IGF-1, remain unclear.

In the present study, we used the serum-free culture system to demonstrate the occurrence of additive crosstalk among the three niche factors that converges into a CD49f/IGF1R-(PI3K/Akt)-Hif-2α signaling loop, to maintain Oct-4 expression and increase cell migration in CD49f^+^AP^+^GSCs. These findings improve our understanding of the regulatory capacity of niche ECM and endocrinal signaling in the proliferation and maintenance of stemness in early embryonic GSCs, and may facilitate the development of potential strategies for engineering stem cell–based cell therapy in regenerative medicine.

## Results

### Laminin Increases the Activity of AP and the Expression of Stemness-Related Genes in Mouse AP^+^GSCs Under IGF-1 Free and Serum-Free Culture Conditions

**FIGURE 1:**
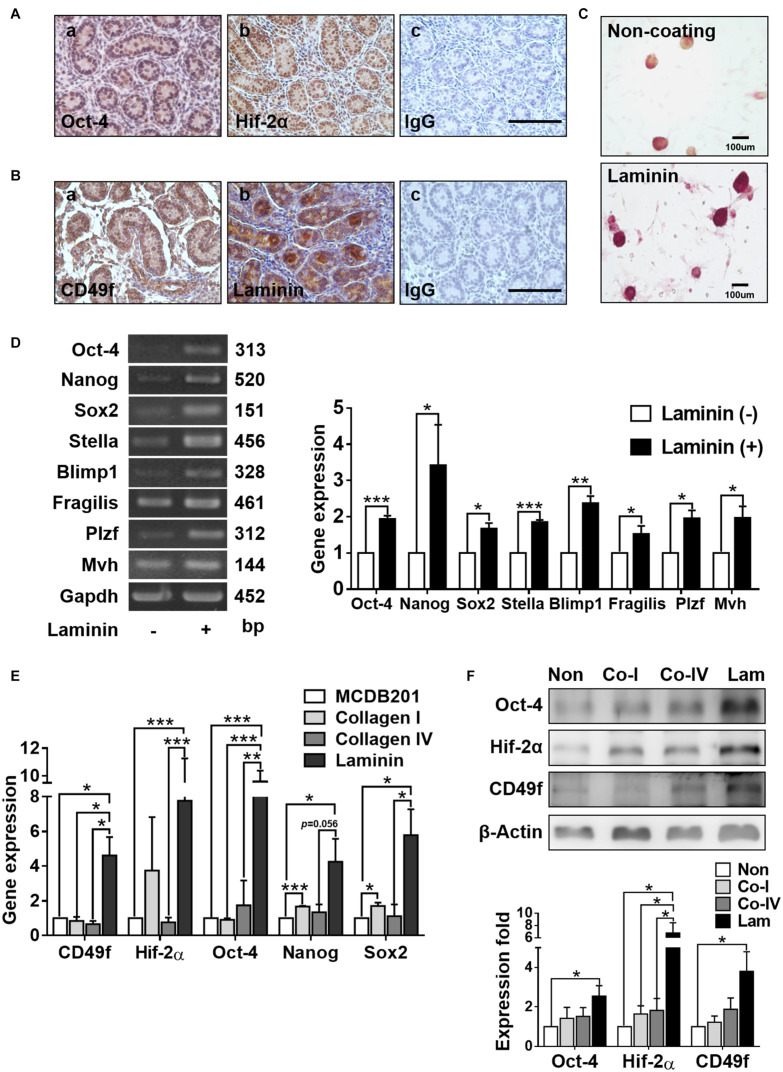
Laminin increases alkaline phosphatase (AP) activity and stemness-related gene expression in mouse germline stem cells (GSCs) (AP^+^GSCs). **(A,B)** Protein expression and localization of Oct-4 **(A-a)**, Hif-2α **(A-b)**, CD49f **(B-a)**, and laminin **(B-b)** in cells obtained from testes of 2 days postpartum mice. **(A-c,B-c)** Control. Bar = 100 μm. **(C)** Stained alkaline phosphatase (AP) activity in GSC colonies cultivated on laminin-coated or non-coated culture plates. Bar = 100 μm. **(D)** Gene expression in GSC colonies cultivated on laminin-coated or non-coated culture plates through reverse transcription polymerase chain reaction (RT-PCR) (left panel) and RT-qPCR (right panel) analyses. **(E)** Gene expression in magnetic-activated cell-sorting system (MACS)-purified CD49f^+^AP^+^GSCs cultivated on culture plates coated with different ECM components determined through RT-PCR analysis. **(F)** Protein expression in MACS-purified CD49f^+^AP^+^GSCs cultivated on culture plates coated with different ECM components through Western blot analysis. Lam: laminin. Co-I: type I collagen. Co-IV: type IV collagen. Statistical data are means ± SEM of at least three independent experiments for each condition. *p < 0.05; **p < 0.01; ***p < 0.001 (Student’s *t*-test).

Neonatal mouse testis (where GSCs reside) harvested on day 2 exhibited positive signals of stemness and hypoxia responses, as manifested in the immunohistochemical staining of Oct-4 and Hif-2α (Figure 1A). The abilities of self-renewal and differentiation of stem cells have been suggested to be associated with the presence of niche ECM factors, such as laminin (Covello et al., 2006). Consistently, we observed 7positive signals of immunostained CD49f in testicular GSCs harvested on day 2, which were surrounded by positive signals of laminin (Figure 1B). The effects of laminin on GSC stemness and hypoxia responses were confirmed by cultivating harvested AP^+^GSCs on laminin-coated or non-coated substrates in serum-free medium (Huang et al., 2014; Kuo et al., 2018). Compared with colony formation of AP^+^GSCs in the absence of laminin, AP^+^GSCs cultivated in the presence of laminin showed obviously a higher AP activity, more cell proliferation (Figure 1C), and significantly higher expressions of stemness-related genes, such as *Oct-4*, *Nanog*, *Sox2*, *Stella*, *Blimp1*, *Fragilis*, *Plzf*, and *Mvh* (Figure 1D).

To further identify the effect of laminin/CD49f signaling on GSC stemness and hypoxia responses, CD49f^+^AP^+^GSCs were isolated using the magnetic-activated cell sorting (MACS, Miltenyi Biotec), where the laminin receptor integrin α6, also known as CD49f, a cell surface marker of AP^+^GSCs was used (Mercier, 2016). The cells were then cultured on substrates coated with various ECM components in serum-free medium. Previously, we found that these MACS-purified CD49f^+^GSCs exhibited high AP activity and expressed pluripotency-related genes, such as *Oct-4*, *Nanog*, *Sox2*, and *Blimp1* (Huang et al., 2014). To mimic the physiological condition, type I collagen, which is enriched in the stroma, and type IV collagen and laminin, the two components enriched in the basement membrane surrounding GSCs *in vivo*, were used as coating materials. Compared with types I and IV collagen, laminin-coated substrates significantly enhanced the expression of CD49f and stemness/hypoxia-related genes at the mRNA (Figure 1E, *Oct-4*, *Nanog*, *Sox2*, and *Hif-2α*) or protein levels (Figure 1F, CD49f, Oct-4, and Hif-2α, the repeated data (*n* = 3) were provided in Supplementary Figure 4).

### IGF-1 Increases the Expression of CD49f Involving IGF-1R-PI3K/Akt-mTOR/Hif-2α Signaling in CD49f^+^AP^+^GSCs

Previously, we showed that niche endocrinal IGF-1 signaling enhanced GSC stemness by upregulating the expression of Hif-2α, which in turn promoted not only Oct-4 expression through direct promoter binding (Huang et al., 2014; Kuo et al., 2018) but also IGF-1 and IGF-1R expression, which led to a self-perpetuating effect on IGF-1 signaling. We also found that the niche hypoxia can activate IGF-1R signaling and enable migration of AP^+^GSCs through a Hif-2α-OCT-4/CXCR4 signaling loop (Huang et al., 2014; Kuo et al., 2018). Hypoxia, endocrinal IGF-1, and ECM laminin are all crucial niche factors for GSC self-renewal and development. Furthermore, both IGF-1 and laminin signaling utilizes the PI3K/Akt pathway (Nguyen et al., 2000; Akeno et al., 2002; Huang et al., 2009, 2014; Yu et al., 2012; Yazlovitskaya et al., 2019). Hence, we could hypothesize that crosstalk exists among signals from these factors.

**FIGURE 2:**
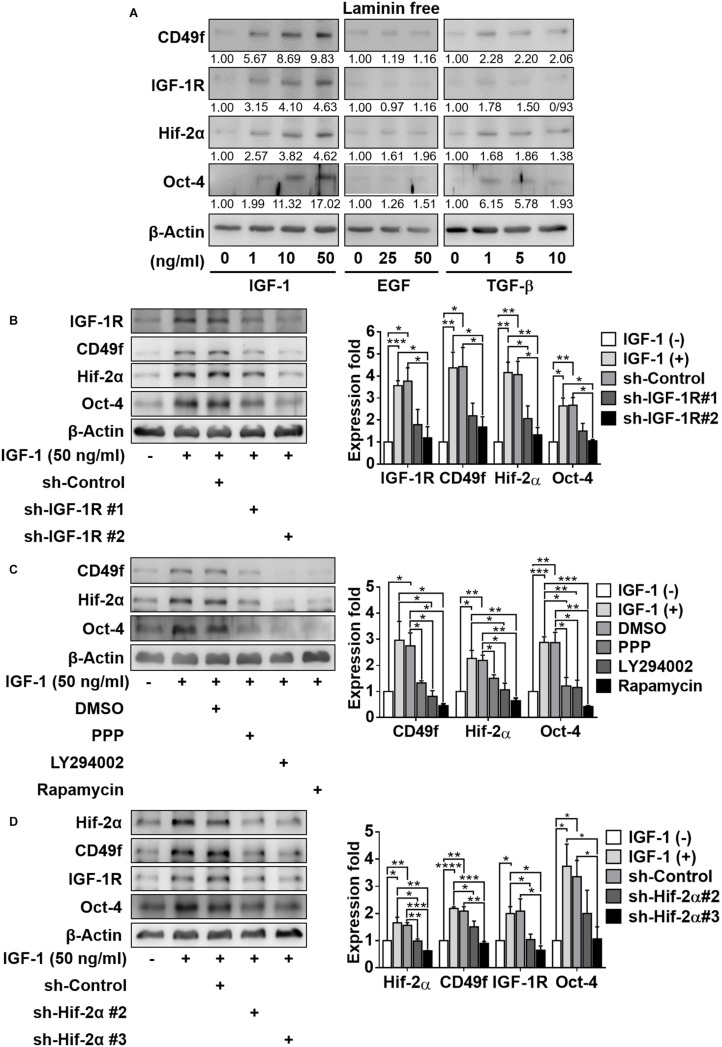
IGF-1 enhances the expression of CD49f, IGF1-R, and Oct-4 involving an IGF1R-PI3K/Akt-mTOR-Hif-2α signaling pathway in CD49f^+^AP^+^GSCs. **(A)** Effect of IGF-1 (0, 1, 10, and 50 ng/ml), EGF (0, 25, and 50 ng/ml), and TGF-β (0, 1, 5, and 10 ng/ml) on expression of CD49f, IGF-1R, Hif-2α, and Oct-4 in CD49f^+^AP^+^GSCs. The relative quantification was normalized to the corresponding β-Actin. **(B)** Protein expression of CD49f, Hif-2α, and Oct-4 in CD49f^+^AP^+^GSCs under IGF-1 treatment (50 ng/ml) with scramble shRNA or shIGF-1R in the absence of laminin. The knockdown efficiencies are given in Supplementary Figure 2. **(C)** Protein expression of CD49f, Hif-2α, and Oct-4 in CD49f^+^AP^+^GSCs under IGF-1 treatment (50 ng/ml) with or without PPP (1 μM), LY294002 (10 μM), or rapamycin (50 nM) in the absence of laminin. **(D)** Effect of shHif-2α on the expression of CD49f, IGF-1R, and Oct-4 in IGF-1-treated CD49f^+^AP^+^GSCs in the absence of laminin. Data are means ± SEM of at least three independent experiments. *p < 0.05; **p < 0.01; ***p < 0.001; ****p < 0.0001 (Student’s *t*-test).

To ascertain the possibility of crosstalk, we first examined whether hypoxia or IGF-1 signaling caused an amplification effect on laminin signaling by upregulating the expression of laminin receptor CD49f. We found that hypoxic culture conditions increased the expression of not only Hif-2α but also IGF-1R and CD49f in CD49f^+^AP^+^GSCs in the absence of IGF-1 and laminin (Supplementary Figure 1). We also found that in the absence of laminin, IGF-1 increased the expression of CD49f, along with IGF-1R, Hif-2α, and Oct-4, in CD49f^+^AP^+^GSCs in a dose-dependent manner; however, no such effect was observed when the cells were treated with epidermal growth factor (EGF) or transforming growth factor beta (TGF-β) (Figure 2A, the original data are provided in Supplementary Figure 5). The specificity of the involvement of IGF-1/IGF-1R signaling in the upregulation of CD49f expression was confirmed by genetic and pharmaceutical manipulation. First, two silencing RNA interference constructs that target endogenous IGF-1R (shIGF-1R) with different knockdown efficiencies were selected for IGF-1R knockdown experiments in CD49f^+^AP^+^GSCs (shIGF-1R#1 and shIGF-1R#2) (Supplementary Figure 2). We found that shIGF-1R#2 effectively suppressed not only the expression of IGF-1R but also the IGF-1-induced expression of CD49f, Oct-4, and Hif-2α in CD49f^+^AP^+^GSCs in the absence of laminin (Figure 2B, the repeated data (*n* = 3) are provided in Supplementary Figure 6). Second, pharmaceutical inhibitors that suppress IGF-1R/PI3K-Akt/mTOR signaling downstream to IGF-1 stimulation, such as cyclolignan picropodophyllin (PPP, a phospho-IGF-1R inhibitor), LY294002 (PI3K/Akt inhibitor), and rapamycin (mTOR inhibitor), were applied to IGF-1-treated CD49f^+^AP^+^GSCs in the absence of laminin. The aforementioned inhibitors also efficiently suppressed IGF-1-induced expression of CD49f, Oct-4, and Hif-2α in CD49f^+^AP^+^GSCs (Figure 2C, the repeated data (*n* = 3) are provided in Supplementary Figure 7).

Our previous study showed that IGF-1-mediated upregulation of IGF-1, IGF-1R, Oct-4, and Hif-2α all depended on the expression of Hif-2α (Huang et al., 2014; Kuo et al., 2018). To ascertain whether this was also applicable to IGF-1-induced CD49f upregulation, we performed genetic perturbation on Hif-2α expression. We used two RNA interference constructs that target endogenous Hif-2α (shHif-2α) with different knockdown efficiencies (#2 and #3). We found that knockdown of Hif-2α (particularly by shHif-2α #3) effectively suppressed not only the expression of Hif-2α but also the IGF-1-induced expression of Oct-4, IGF-1R, and CD49f (Figure 2D, the repeated data (*n* = 3) are provided in Supplementary Figure 8). These results suggest the crosstalk among laminin, hypoxia, and IGF-1 signaling and that PI3K/Akt-mTOR-Hif-2α signaling is involved in the IGF-1-mediated upregulation of CD49f expression in CD49f^+^AP^+^GSCs.

### IGF-1 and Laminin Additively Increase the Expression of IGF-1R, CD49f, Hif-2α, and Oct-4 in CD49f^+^AP^+^GSCs

**FIGURE 3:**
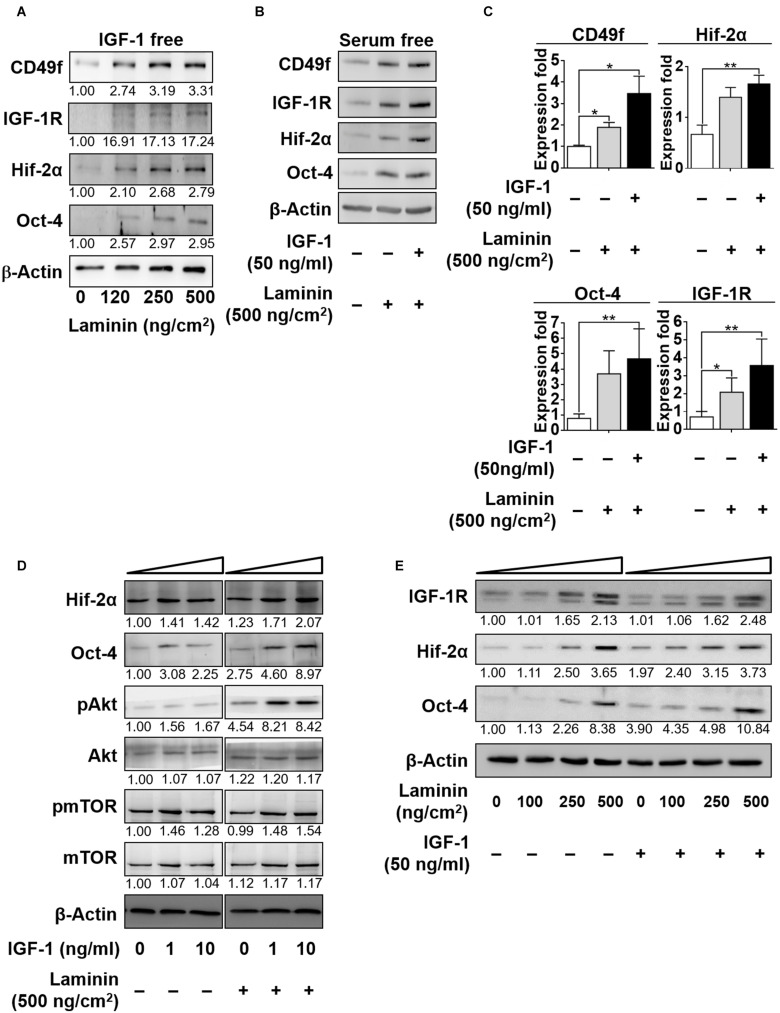
IGF-1 and laminin act additively to increase the expression of CD49f, IGF-1R, Hif-2α, and Oct-4 in CD49f^+^AP^+^GSCs. **(A)** Dose-dependent effects of laminin (0, 120, 250, and 500 ng/cm^2^) on the protein expression of CD49f, IGF-1R, Hif-2α and Oct-4 in CD49f^+^AP^+^GSCs in the absence of IGF-1. **(B)** Effects of laminin (500 ng/cm^2^) with or without IGF-1 (50 ng/ml) treatment on the expressions of IGF-1R, CD49f, Hif-2α, and Oct-4. **(C)** Quantitative and statistical analysis of the effects on **(B)**. Data are means ± SEM of at least three independent experiments. *p < 0.05; **p < 0.01 (one-way ANOVA). **(D)** Dose-dependent effects of IGF-1 (0, 1, and 10 ng/ml) with or without laminin (500 ng/cm^2^) treatment on the protein expression of Hif-2α, Oct-4, and on the Akt-mTOR signaling. **(E)** Dose-dependent effects of laminin (0, 100, 250, and 500 ng/cm^2^) with or without IGF-1 (50 ng/ml) treatment on the expression of IGF-1R, Hif-2α, and Oct-4. The relative quantification was normalized to the corresponding β-actin.

Robust control in GSC self-renewal and differentiation requires synergistic coordination in the responses to niche factors. Having shown that IGF-1 upregulated the expression of laminin receptor CD49f, we examined whether exposure to laminin reciprocally affected IGF-1 signaling. IGF-1 and laminin signaling have both been reported to activate the PI3K/Akt pathway (Nguyen et al., 2000; Akeno et al., 2002; Huang et al., 2009, 2014; Yu et al., 2012; Yazlovitskaya et al., 2019), indicating a potential synergy in their signaling. To examine this possibility, CD49^+^AP^+^GSCs were cultivated in serum-free culture medium in the presence or absence of laminin and with or without IGF-1 in the medium. Figure 3A shows that in the absence of IGF-1, laminin enhanced the expression of not only CD49f but also that of IGF-1R, Oct-4, and Hif-2α in a dose-dependent manner and the repeated data (*n* = 3) are provided in Supplementary Figure 9. An additive enhancement of the expression of IGF-1R, CDC49f, Hif-2α, and Oct-4 in CD49f^+^AP^+^GSCs was observed when cells were cotreated with laminin (500 ng/cm^2^) and IGF-1 (50 ng/ml). Cotreatment resulted in higher expression than did treating the cells with each of the factors alone, despite that the enhancement was weaker in Hif-2α and Oct-4 expressions (Figures 3B,C, the repeated data (*n* = 3) are provided in Supplementary Figure 10). In addition, the IGF-1-induced upregulation of Akt/mTOR activity and Oct-4/Hif-2α expression appeared in a dose-dependent manner in the presence of laminin (Figure 3D, the original data and repeated data are provided in Supplementary Figure 11). Likewise, the laminin-induced upregulation of expressions of IGF-1R, Oct-4, and Hif-2α exhibited a dose-dependent response to IGF-1 (Figure 3E, the original data were provided in Supplementary Figure 12). These data suggest that the effects of IGF-1 and laminin on the upregulation of IGF-1R, CD49f, Hif-2α, and Oct-4 expression are additive.

### Both IGF-1R and CD49f Are Required for IGF-1/Laminin-Mediated Additive Upregulation of CD49f, IGF-1R, Oct-4, and Hif-2α Expression in CD49f^+^AP^+^GSCs

**FIGURE 4:**
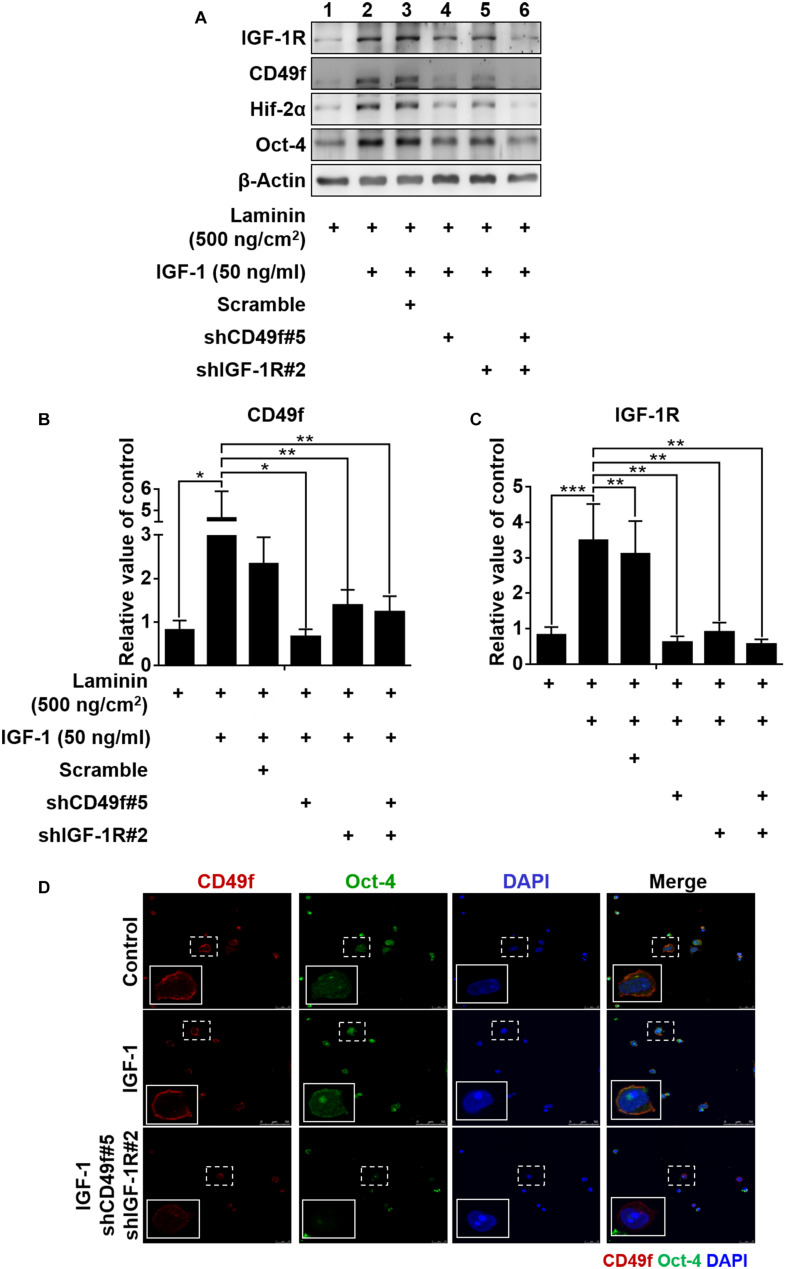
Both IGF-1R and CD49f are required for IGF-1/laminin-mediated additive upregulation of CD49f, IGF-1R, Hif-2α, and Oct-4 expression in CD49f^+^AP^+^GSCs. **(A)** Suppression effects of shCD49f and/or shIGF-1R on the expression of Hif-2α, Oct-4, CD49f, and IGF-1R in CD49f^+^AP^+^GSCs treated with IGF-1 (50 ng/ml) on laminin-coated substrates (500 ng/cm^2^). **(B,C)** The quantitative and statistical analyses of shCD49f- and/or shIGF-1R-mediated suppressing effects on the expression of **(B)** CD49f and **(C)** IGF-1R from **(A)**. Data are means ± SEM of at least three independent experiments. *p < 0.05; **p < 0.01, ***p < 0.001 (one-way ANOVA). **(D)** The suppressing effect of shCD49f and shIGF-1R on cellular localization of immunocytochemistry stained Hif-2α and Oct-4 in CD49f^+^AP^+^GSCs treated with IGF-1 (50 ng/ml) on laminin-coated culture plates (500 ng/cm^2^).

To confirm the specificity of the involvement of CD49f/IGF-1R in the laminin/IGF-1-mediated effect, constructs of silencing RNA interference that target endogenous CD49f (shCD49f) or IGF-1Rβ (shIGF-1R) were engineered and applied to CD49f^+^AP^+^GSCs cotreated with laminin (500°ng/cm^2^) and IGF-1 (50°ng/ml). Five constructs for each target were engineered. Among them, we selected those with the highest knockdown efficiencies, shCD49f#5 for CD49f and shIGF-1R#2 for IGF-1Rβ (Figure 4A, the repeated data (*n* = 3) are provided in Supplementary Figure 13). Cotreatment with laminin and IGF-1 significantly increased the expression of CD49f, IGF-1R, Oct-4, and Hif-2α in CD49f^+^AP^+^GSCs compared with that in cells treated with laminin alone (Figure 4B, lane 2 vs. lane 1, and Figure 4C, bar 2 vs. bar 1); however, the addition of shCD49f#5 or shIGF-1R#2 effectively suppressed the effect of cotreatment on Hif-2α, Oct-4, CD49f, and IGF-1R expression (Figure 4B, lane 4–6 vs. lane 3, and Figure 4C, bar 4–6 vs. bar 3). Figure 4D shows the corresponding confocal images of Oct-4 and CD49f stained through immunocytochemistry. Given the activation of the PI3K-Akt pathway in laminin and IGF-1 signaling (Nguyen et al., 2000; Akeno et al., 2002; Huang et al., 2009, 2014; Yu et al., 2012; Yazlovitskaya et al., 2019) and the dependence of IGF-1 signaling on Hif-2α expression (Huang et al., 2014; Kuo et al., 2018), the results suggest the presence of additive crosstalk in IGF-1/laminin signaling that acts through a CD49f/IGF1R-(PI3K/Akt)-Hif-2α signaling loop, which in turn regulates the maintenance of Oct-4 expression of mouse CD49f^+^AP^+^GSCs.

### Laminin Induces Cell Morphological Changes and Genetic Reprogramming

**FIGURE 5:**
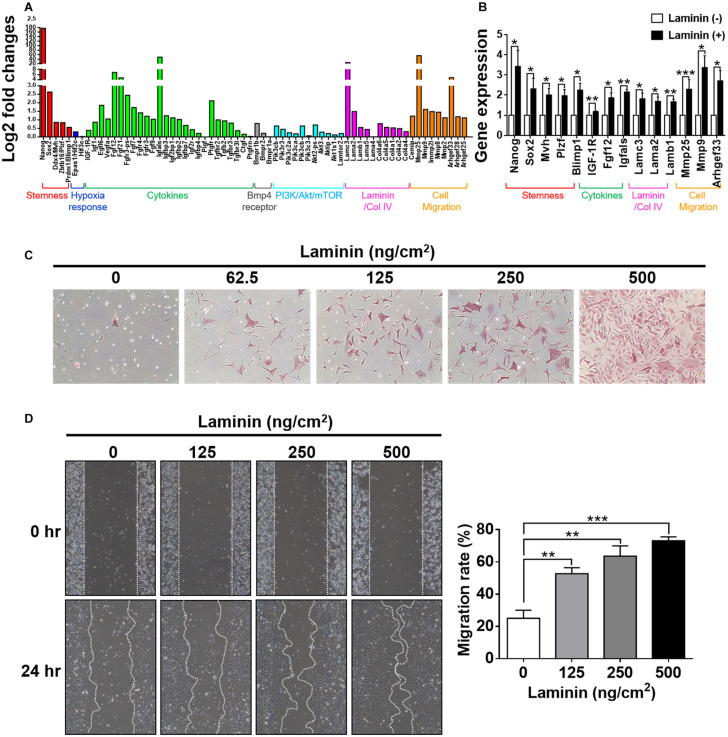
Laminin induces morphological changes and genetic reprogramming. **(A)** Analyses of RNA-seq data collected from CD49f^+^AP^+^GSCs cultivated on laminin-coated and non-coated substrates. Genes were grouped into color categories. Red: stemness. Blue: hypoxia responses. Green: cytokines. Black: BMP4 receptors. Cyan: PI3K/Akt/mTOR pathway. Pink: basement membrane components. Orange: cell migration. **(B)** Validation of RNA-seq data in **(A)** by RT-qPCR. Data are means ± SEM of at least three independent experiments. *p < 0.05; **p < 0.01; ***p < 0.001 (Student’s *t*-test). **(C)** Dose-dependent effects of laminin on cell morphology and alkaline phosphatase (AP) activity. The intensity of the red stain indicates AP activity. **(D)** Wound-healing assay showed that laminin coating enhanced cell migration in a dose-dependent manner. Data are means ± SEM of at least three independent experiments. **p < 0.01; ***p < 0.001 (one-way ANOVA).

The development of germ cells begins with the specification of PGCs marked by the expression of transcription factors, such as Fragilis, Stella, and Blimp1 (Saitou et al., 2002; Ohinata et al., 2005), following which the cells acquire the ability to signal to external BMP4. BMP4 enables PGC competence (Lawson et al., 1999; Ying et al., 2000, 2001); this is the ability to facilitate niche development by secreting the basement membrane and paracrine components as well as the ability to migrate into the genital ridge where PGCs become mature germ cells. In the present work, we showed that laminin induced the upregulation of *Fragilis*, *Stella*, and *Blimp1* in CD49f^+^AP^+^GSCs (Figure 1D), as well as the upregulation of CD49f and IGF1-R (Figure 3A). To determine whether exposure to laminin enhanced the ability of CD49f^+^AP^+^GSCs to signal to BMP4, to participate in niche development, or to migrate, we performed RNA sequencing on CD49f^+^AP^+^GSCs cultivated in the presence or absence of laminin (500°ng/cm^2^) in serum-free culture medium. Although our serum-free CD49f^+^AP^+^GSC model system served as a convenient platform to study the interplay of niche factor–mediated signaling *in vitro*, long-term cultivation on tissue-culture plates can problematically cause significant distortion of genotype and phenotype. Thus, we harvested the cells for RNA sequencing after 3 days of cultivation. The data were analyzed using fold change (i.e., log_2_) between the laminin-treated and untreated samples (Figure 5A). The results showed that exposure to laminin induced the maintenance of stemness, hypoxia response, and PI3K/Akt/mTOR signaling to some degree (Figure 5A, red, blue, and cyan, respectively). In addition, laminin increased the expression of receptor subunits for BMP4 signaling (Figure 5A, gray), the secretion of cytokines and basement membrane components (Figure 5A, green and pink), and the expression of molecules involved in cell migration (Figure 5A, orange). Upregulation of these genes were confirmed by RT-qPCR analysis (Figure 5B).

To ascertain whether these laminin-induced genetic changes reprogrammed cell behavior, we performed cell-based phenotypic assays. First, we examined how cells changed their morphology in response to the exposure of laminin. By cultivating CD49f^+^AP^+^GSCs on substrates coated with various concentrations of laminin, we found that laminin enhanced cell spreading, protruding, and AP activity in a dose-dependent manner (Figure 5C). Next, we used wound-healing assay to examine how cells modulated their migration in response to laminin. The results showed that laminin enhanced cell migration in a dose-dependent manner (Figure 5D). Of note, none of these changes was related to cell proliferation as quantitative measurements on cell growth rates showed a laminin-independent trend (Supplementary Figure 3). Taken together, these results suggested that the exposure to laminin reprogrammed the ability of CD49f^+^AP^+^GSCs to migrate, affected the assembly of the microenvironment, and maintained stemness, hypoxia response, and PI3K/Akt/mTOR signaling.

## Discussion

**FIGURE 6:**
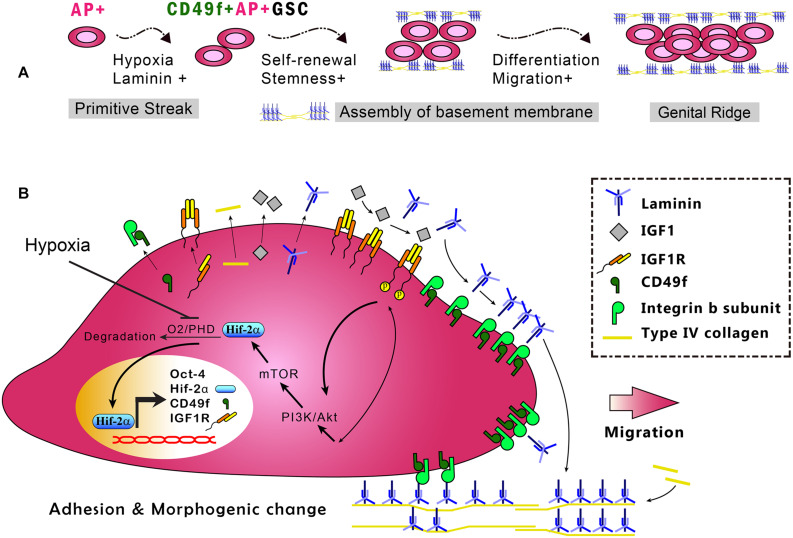
*In vitro* model of the CD49f/IGF1R-Hif-2α regulatory circuit in CD49f^+^AP^+^GSCs. **(A)** Depiction of the stages in early germ cell development. **(B)** *In vitro* model of CD49f/IGF1R-Hif-2α signaling circuit in mouse CD49f^+^AP^+^GSCs under hypoxic conditions. AP, alkaline phosphatase; HIF, hypoxia-inducible protein; IGF-1, insulin-like growth factor-1; IGF-1R, insulin-like growth factor-1 receptor; PHD, prolyl hydroxylases. Notably, the laminin lattice network can be assembled on the cell surface through integrin and in turn be incorporated into the underlying type IV collagen network, thereby forming the basement membrane (Hohenester and Yurchenco, 2013; Yurchenco, 2015).

The fate of stem cells, including self-renewal and differentiation abilities, is regulated by the interplay of internal gene expression and external mechanochemical signals from the niche. In GSCs, development involves maintaining the ability of self-renewal and acquiring the ability to migrate into the genital ridge where the cells mature (Figure 6A). Examples of niche factors include oxygen tension, endocrinal factors, and ECM, all of which can signal to each other, resulting in a complex niche signaling network. For decades, the understanding of the niche signaling network has been hindered by the lack of proper *in vitro* cell model systems. This hindrance in GSC research has recently been resolved by our novel serum-free culture system. By using our serum-free culture system, we showed that niche hypoxia can activate the niche endocrinal IGF-1 signaling to maintain Oct-4 expression, promote symmetric self-renewal, and enable migration of AP^+^GSCs through the Hif-2α-OCT-4/CXCR4 signaling loop (Huang et al., 2014; Kuo et al., 2018). That work highlighted the connection between two crucial niche factors, namely, hypoxia and endocrinal factors. However, the coordination of other critical niche factors, such as ECM, with hypoxia and endocrinal signals to maintain Oct-4 expression in AP^+^GSCs remains unknown. Here, using CD49f^+^AP^+^GSCs as the model system, we advanced our previous findings by showing that niche hypoxia increases the expression of not only IGF-1R but also the laminin receptor CD49f. We also found an additive effect in CD49f/IGF1R-(PI3K/Akt)-Hif-2α signaling for the maintenance of Oct-4, and that exposure to niche ECM laminin results in genetic reprogramming of cell migration ability (Figures 5B, 6). In the present study, we unveiled the niche-signaling network between ECM (laminin) and hypoxia–endocrinal signaling (IGF1-Hif-2α) in the embryonic development of CD49f^+^AP^+^GSCs.

Hypoxia (i.e., low oxygen tension) is a physiological condition that appears during early embryogenesis. It occurs predominately in tissues undergoing rapid growth and has been shown to promote the survival of stem cells, including human ES cells (Ezashi et al., 2005), PGCs (Scortegagna et al., 2005; Covello et al., 2006), induced pluripotent stem cells (iPSCs) (Yoshida et al., 2009), hematopoietic stem cells (Danet et al., 2003), and neural crest stem cells (Morrison et al., 2000; Studer et al., 2000). Hif-2α has been associated with early PGC development (Covello et al., 2006). When PGC cells migrate from the hindgut to the genital ridge, they maintain their Oct-4 levels and AP activity and increase their cell number from 50 (E8.5 embryos) to 20,000 (E12.5 genital ridges) (Covello et al., 2006). Experiments with transgenic mice have shown that the loss of Hif-2α (Hif-2α^–/–^) severely reduced the number of PGCs from 20,000 to approximately 20 in E12.5 genital ridges (Covello et al., 2006) and caused azoospermia (Scortegagna et al., 2005). As Hif-2α directly regulates Oct-4 (Covello et al., 2006) and c-Myc (Keith and Simon, 2007) expression, we can reasonably assume that regulating Hif-2α expression can affect Oct-4 expression and stem cell proliferation.

In support of the aforementioned assumption, we have previously shown that hypoxia elevates the expression of stemness-related genes (e.g., *Oct-4*, *Sox2*, *Nanog*, and *Klf-4*) in mouse AP^+^GSCs. In particular, hypoxia can increase the expression of IGF-1/IGF-1R, which in turn stimulates the expression of Hif-2α (Huang et al., 2014) and leads to a feedforward loop between hypoxia and IGF-1 signaling. Herein, we additionally showed that Hif-2α knockdown suppressed IGF-1-induced IGF-1R/CD49f expression in CD49f^+^AP^+^GSCs (Figure 2D). Consistent with our results, IGF-1/IGF-1R signaling was reported to be associated with Hif-1α or Hif-2α expression in cancers and in cells of somatic lineage (Akeno et al., 2002; Carroll and Ashcroft, 2006; Catrina et al., 2006). Similarly, human cancer converged at the Hif-2α oncogenic axis has been suggested to require IGF-1R activation (Franovic et al., 2009). Thus, the crosstalk between hypoxia and IGF-1/IGF-1R signaling is manifested not only in the GSC development and maintenance but also in tumorigenesis (Lee et al., 2019). Identifying the molecular mechanisms underlying such crosstalk will not only benefit stem cell–based cell therapy for regenerative medicine but also provide insights into cancer biology and therapy.

ECM components (such as laminin) are the important niche factors in the microenvironment surrounding GSCs (Figure 1B). An increasing body of evidence has shown that niche laminin is highly associated with the development of embryonic germ cells and cancer. In stem cell development, for example, laminin was demonstrated to promote embryonic GSC migration—a significant amount of laminin receptor CD49f was found to be expressed in these cells (Hedger, 1997; Morita-Fujimura et al., 2009). Laminin/CD49f activation has also been shown to regulate Oct-4 expression through PI3K/Akt signaling in human mesenchymal stem cells (Yu et al., 2012). Consistently, laminin has been reported to be a potent substrate for large-scale expansion of induced pluripotent stem cells of humans (Paccola Mesquita et al., 2019). Remodeling of basal lamina at the niche of skeletal muscle stem cells was also found to mediate stem cell self-renewal, and genetic ablation of laminin-α1, disruption of integrin-α6 signaling, or the blockade of matrix metalloproteinase activity can impair satellite cell expansion and self-renewal (Rayagiri et al., 2018). In patients with somatic cancers, by contrast, cancer cells expressing a high level of laminin and CD49f are associated with poor prognosis, high recurrence rates, and high incidence rates of cancer stem cells (Chang et al., 2012; Vieira et al., 2014), and the blockade of a laminin-411–Notch axis was shown to inhibit glioblastoma growth through tumor–microenvironment crosstalk (Sun et al., 2019). Nevertheless, how laminin signaling is coupled with the signals from other niche factors, such as hypoxia and IGF-1, both of which play crucial roles in cancer and stem cell development, remains largely unknown.

Here, we showed that niche hypoxia upregulates the expression of not only IGF-1R but also the laminin receptor CD49f (Supplementary Figure 1) and that Hif-2α is involved in IGF-1-induced upregulation of CD49f (Figure 2D), while autocrine signals in IGF-1/IGF-1R and laminin/CD49f activation increased Hif-2α expression (Figures 1–4). These results provide evidence suggesting that the presence of crosstalk and a signaling network among the three niche factors, namely, hypoxia, endocrinal IGF-1, and ECM laminin, that can act additively through a CD49f/IGF1R-Hif-2α signaling loop to maintain Oct-4 expression in the embryonic CD49f^+^AP^+^GSCs.

In the present study, Oct-4 expression level was primarily used as the reporter to indicate the extent of stemness. Consistent with our results, the level of Oct-4 expression *in vivo* was closely associated with cell fate specification during embryogenesis (Sun et al., 2019). In male GSCs, for example, Oct-4 expression levels are different among PGCs, postmigratory PGCs (i.e., gonocytes), and spermatogonial stem cells (Pesce et al., 1998); furthermore, the importance of Oct-4 expression level in PGC cell fate specification has been documented using a conditional *Cre*/*loxP* gene-targeting strategy (Kehler et al., 2004). To regulate Oct-4 expression levels, several regulators have been identified, including Hif-2α (Covello et al., 2006), EpCAM (Huang et al., 2011), estrogen (Zhang et al., 2008; Jung et al., 2011), SUMO1/sentrin-specific peptidase proteins (Wu et al., 2012), and IGF-1/IGF-1R signaling (Bendall et al., 2007; Huang et al., 2009). Among these regulators, Hif-2α is well-documented because hypoxia is a common niche factor in embryonic stem cell development. One factor that has not been previously reported but was reported in the present study is laminin. In addition to laminin, IGF-1 signaling is of particular interest because its role in modulating the proliferation and pluripotency of stem cells has been extensively studied (Huang et al., 2009, 2014; Li and Geng, 2010). For example, studies have reported that IGF-1 cooperates with basic fibroblast growth factor to maintain self-renewal in human embryonic stem cells (Bendall et al., 2007; Huang et al., 2014). In parallel, the PI3K/Akt signaling axis, which is downstream to both IGF-1 and laminin signaling, has been reported to be involved in crosstalk with self-renewal mechanisms in embryonic stem cells (Watanabe et al., 2006; McLean et al., 2007). In the context of cancer, the activation of IGF-1R signaling has been demonstrated to initiate the expression of stemness in breast cancer (Motallebnezhad et al., 2016) and hepatocellular carcinomas (HCC) (Chang et al., 2015, 2016). Consistent with these reports, our previous data showed that the activation of the PI3K/Akt pathway can promote PGC proliferation and maintain Oct-4 expression in embryonic germ cells (Kimura et al., 2003; Moe-Behrens et al., 2003; Kuo et al., 2018) and that niche IL-6/IGF-IR signaling causes poor prognosis of hepatitis B virus (HBV)-related HCC because of Oct-4/Nanog expression (Chang et al., 2015).

In summary, this study used primary CD49f^+^AP^+^GSCs and serum-free culture medium and provided the first evidence demonstrating the enhanced expression of CD49f and IGF-1R because of hypoxia and the cooperation of niche laminin with the associated CD49f/IGF1R-(PI3K/Akt-mTOR)-Hif-2α signaling loop to maintain cell proliferation and Oct-4 expression in embryonic CD49f^+^AP^+^GSCs (Figure 6). Findings from this study provide important insights into the niche signaling network among ECM laminin/CD49f signaling, endocrinal IGF-1/IGF-1R signaling, and hypoxia responses in early embryonic germ cell development. These insights have potential applications for future strategies to engineer cell therapy for regenerative medicine.

## Experimental Procedures

### Electroporation of Short Hairpin RNA

The shControl (TRCN0000072246), shIGF-1R#1 (TRCN0000023489), shIGF-1R#2 (TRCN0000023491), shCD49f#3 (TRCN0000066150), and shCD49f#5 (TRCN0000066152) plasmids were purchased from National RNAi Core (Taiwan). Double-stranded hairpin oligonucleotides designed to target the mouse Hif-2α cDNA (NM_010137) at the sequence position 2052–2070 (5′-GATGAGGTCTGCAAAGGAC-3′, shRNA#2) and 87–105 (5′-GGAGACGGAGGTCTTCTAT-3′, shRNA#3) of the Hif-2α gene were cloned into the *Bam*HI/*Not*I site of the pGSH1-GFP vector to generate shHif-2α. Gene knocked-down GSCs were generated through electroporation with plasmids (15 μg). Electroporation was performed using an electroporator (BTX) at 250 V for three pulses, each pulse lasting 0.1 ms with an interval of 0.25 s.

### RNA Isolation and Reverse-Transcription Polymerase Chain Reaction

The AP^+^GSC colonies and the MACS-purified CD49f^+^GSCs were collected, and the total RNA was extracted using an RNeasy Micro Kit (QIAGEN, Valencia, CA, United States) according to the manufacturer’s instructions. Three micrograms of total RNA and a random primer (Invitrogen, Carlsbad, CA, United States) were used to synthesize complementary (c)DNA. The cDNA synthesis was performed at 42°C for 50 min in a final volume of 20 μl according to the manufacturer’s instructions for Superscript III reverse transcriptase (Invitrogen). Polymerase chain reaction (PCR) was performed with PlatinumTaq polymerase (Invitrogen), and the real-time RT-qPCR amplicons were titrated within a linear range of amplification. The accession numbers, primer pair sequences, and annealing temperatures are listed in Supplementary Table 1. Beta-2 microglobin was used as an internal control. PCR products were separated through agarose gel electrophoresis, and the DNA bands were visualized using ethidium bromide under ultraviolet light. RT-qPCR analysis of at least three independent cultures was performed for all experiments.

### Western Blotting Analysis

The MACS-purified CD49f^+^GSCs were collected and lysed in reducing 2× Laemmli sample buffer, subjected to 10% SDS-PAGE, and then transferred to a polyvinylidene difluoride (PVDF) membrane for Western blot analysis. The primary antibodies used in the experiment are listed in Supplementary Table 2, and horseradish peroxidase (HRP)-conjugated anti-mouse/rabbit immunoglobulin G (IgG; 1:2,000) was used as the secondary antibody. The activity of HRP was detected using an enhanced chemiluminescence system according to the manufacturer’s instructions (Amersham Pharmacia Biotech., Buckinghamshire, United Kingdom). Quantifications of the protein bands were performed using the SPOT DENSO software on an AlphaImager2200 instrument (Alpha Innotech Corporation, CA, United States).

### RNA Sequencing Analysis

The MACS-purified CD49f^+^GSCs cultivated on laminin-coated and non-coated substrates in the serum-free medium were harvested. RNA sequencing (RNA-seq) was performed by the Phalanx Biotech Group (Taipei, Taiwan). Briefly, RNA samples from the cell lysates were first enriched in mRNA by using oligo(dT) beads. Subsequently, double-stranded cDNA synthesis, end repair, the addition of the nucleotide “A” overhangs and adaptors, cDNA second-strand degradation, fragment selection, PCR amplification, library quality test, and illumine sequencing were performed. For sequencing, the amplified raw output was trimmed to a 150-bp fragment, and the cutoff was applied when the sliding window (four-base window) dropped below 15 or when the read was shorter than 35 base pair. This method yielded a total of 20 million reads. For sequence alignment, STAR was used to map preprocessed read data with the reference genome GRCm38.p6. After the reads were aligned to the genome, the package Cuffquant was used on the resulting alignment files to compute gene and transcript expression profiles. Cuffdiff, a module of the Cufflinks package, merged assemblies from two or more conditions to estimate the expression levels by calculating the number of RNA-seq fragments per kilobase of transcript per million (FPKM) fragments mapped. Cuffdiff was used to test the statistical significance of observed changes and identify genes that were regulated at the transcriptional or posttranscriptional level. Clustering analysis was performed to segregate upregulated and downregulated genes in the laminin-treated and non-laminin-treated samples. The differentially expressed genes in the laminin-treated and non-treated samples were then distributed according to fold change (i.e., log_2_) and significance (i.e., *p*- and *Q*-values).

### Statistical Analysis

All experiments were repeated at least three times with individual samples. Data were expressed as the mean ± standard error of the mean (SEM). Differences in means were assessed using the *t*-test or one-way analysis of variance analysis (ANOVA) and *post-hoc* tests (GraphPad InStat 3.0, GraphPad Software, Inc., La Jolla, CA, United States). A *p*-value of < 0.05 was considered statistically significant.

## Data Availability Statement

The original contributions presented in the study are included in the article/Supplementary Material, further inquiries can be directed to the corresponding author/s.

## Ethics Statement

The animal study protocol was approved by the Institutional Animal Care and Use Committee or Panel (IACUC/IACUP) at Taipei Medical University (Affidavit of Approval of Animal Use Protocol # LAC-2017-0532).

## Author Contributions

H-KA, S-WP, and C-CL: conception and design of the study, collection and organization of data, data analysis, and data interpretation. Y-CK, T-YLa, and Y-LW: collection and organization of data. H-NH: administrative and final approval of the manuscript. C-LG: RNA-seq data analysis, graphical summary, and manuscript writing. T-YLi and Y-HH: conception and design of the study, data analysis and interpretation, manuscript writing, and final approval of the manuscript. All authors contributed to the article and approved the submitted version.

## Conflict of Interest

The authors declare that the research was conducted in the absence of any commercial or financial relationships that could be construed as a potential conflict of interest.

## Publisher’s Note

All claims expressed in this article are solely those of the authors and do not necessarily represent those of their affiliated organizations, or those of the publisher, the editors and the reviewers. Any product that may be evaluated in this article, or claim that may be made by its manufacturer, is not guaranteed or endorsed by the publisher.

## Abbreviations

AP: alkaline phosphatase
GSCs: germline stem cells
Hif-2α: hypoxia-inducible factor 2 alpha
IGF-1: insulin-like growth factor 1
IGF-1R: insulin-like growth factor 1 receptor
PI3K: phosphatidylinositol 3-kinase
PGC: primordial germ cell.
