# Supplementary material for: Niche Laminin and IGF-1 Additively Coordinate the Maintenance of Oct-4 Through CD49f/IGF-1R-Hif-2α Feedforward Loop in Mouse Germline Stem Cells
Source: Front Cell Dev Biol. 2021 Jul 26;9:646644. doi: 10.3389/fcell.2021.646644 (PMC8351907; doi:10.3389/fcell.2021.646644)
Supplement: Supplementary file 1 [file Data_Sheet_1.doc]

**Supplemental information**

**Supplemental Figures**

**
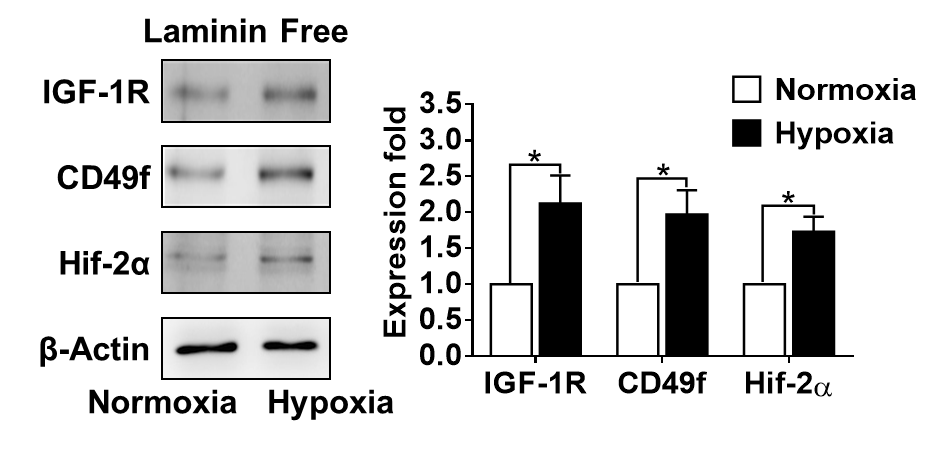
**

**Figure S1: Effect of oxygen tension on the expression levels of IGF-IR, CD49f, and Hif-2α.** CD49f^+^AP^+^GSCs were cultivated in laminin-free medium, and incubated under normoxic (21% O_2_) or hypoxic condition (5% O_2_) for 2 days. β-Actin serves as an internal control. The statistics were shown on the right. Data are means ± standard error of the mean (SEM) from at least 3 independent experiments. * indicates *P* < 0.05 (Student’s *t* test).

**
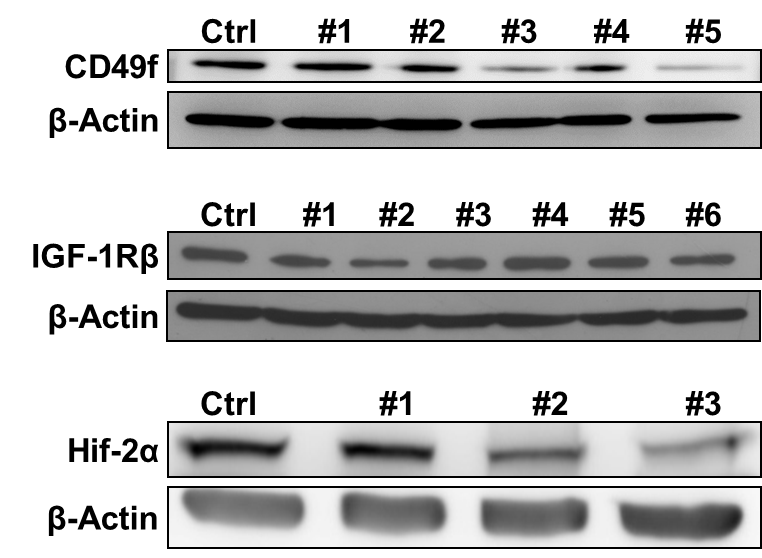
**

**Figure S2: Suppression effects of silencing RNA constructs targeting endogenous CD49f, IGF1-Rβ, and Hif-2α.** CD49f^+^AP^+^GSCs were electroporated with various short hairpin oligonucleotide sequence constructs to target CD49f (A), IGF1-Rβ (B), or Hif-2α (C) (see Methods for details). Ctrl stands for the control construct shControl (TRCN0000072246), while #1−#6 indicates the label of constructs.


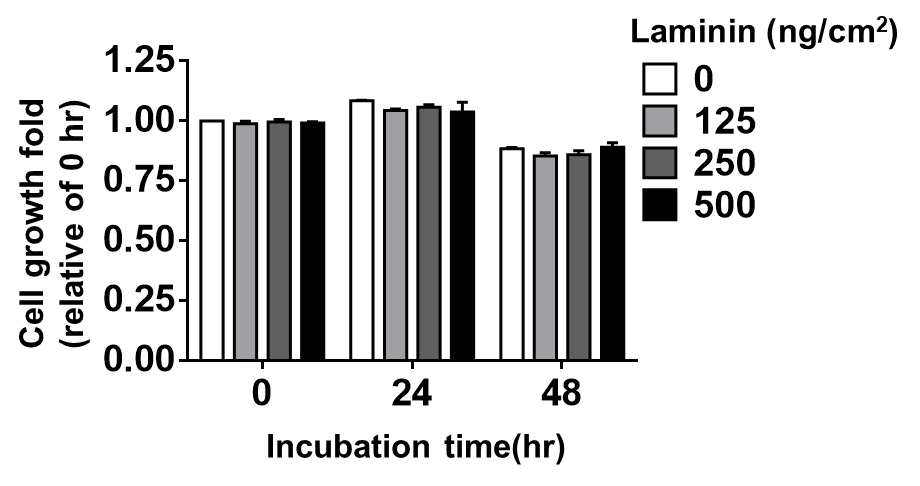


**Figure S3: Identification of cell proliferation fold of CD49f^+^AP^+^GSCs at different laminin (LN)-coated concentrations.** Values of growth folds were normalized to the measurement at 0h. The coating concentrations of laminin (LN, ng/cm^2^) were indicated on the right. Data were means ± SEM from at least 3 independent experiments.

**
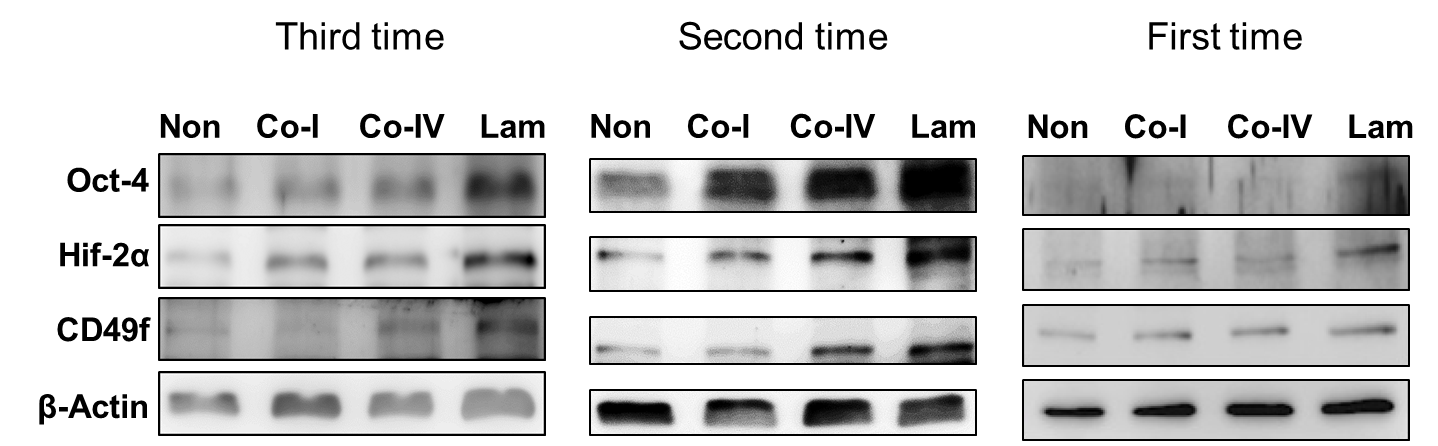
**

**Figure S4: The repeated three times data of Figure 1F.** Protein expression in MACS-purified CD49f^+^AP^+^GSCs cultivated on culture plates coated with different ECM components through Western blot analysis. Lam: laminin. Co-I: type I collagen. Co-IV: type IV collagen.

**
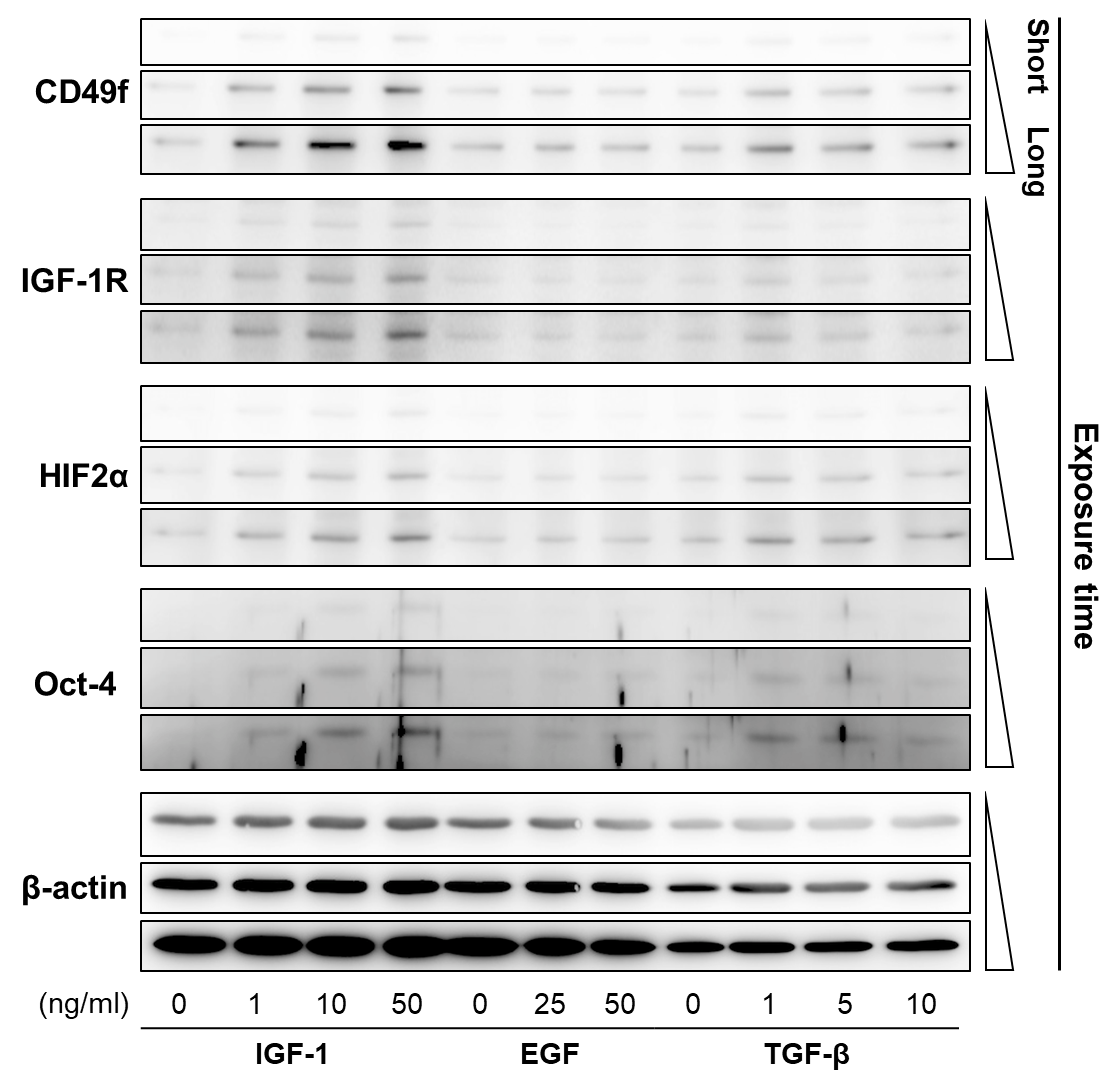
**

**Figure S5: The original western blot data of Figure 2A with different exposure times.** Effect of IGF-1 (0, 1, 10, and 50 ng/mL), EGF (0, 25, and 50 ng/mL), and TGF-β (0, 1, 5, and 10 ng/mL) on expression of CD49f, IGF-1R, Hif-2α, and Oct-4 in CD49f^+^AP^+^GSCs.

**
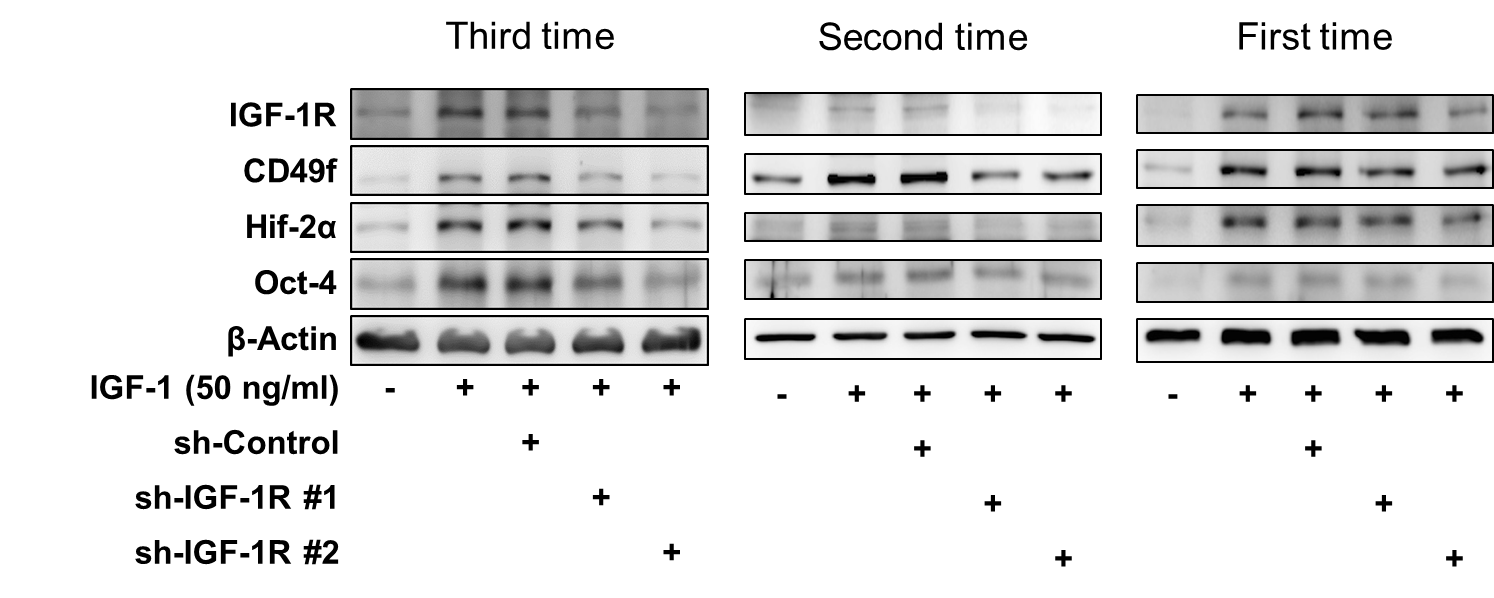
**

**Figure S6: The repeated three times data of Figure 2B.** Protein expression of CD49f, Hif-2α, and Oct-4 in CD49f^+^AP^+^GSCs under IGF-1 treatment (50 ng/mL) with scramble shRNA or shIGF-1R in the absence of laminin.

**
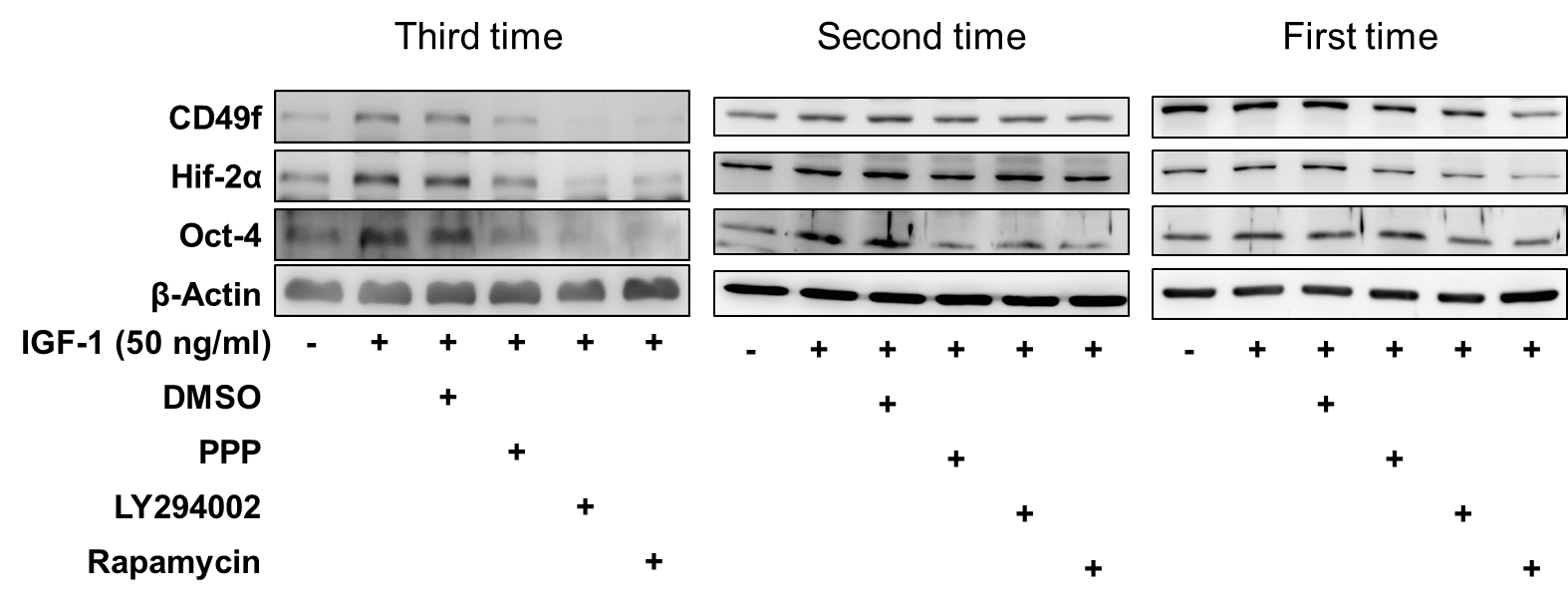
**

**Figure S7: The repeated three times data of Figure 2C.** Protein expression of CD49f, Hif-2α, and Oct-4 in CD49f^+^AP^+^GSCs under IGF-1 treatment (50 ng/mL) with or without PPP (1 μM), LY294002 (10 μM), or rapamycin (50 nM) in the absence of laminin.

**
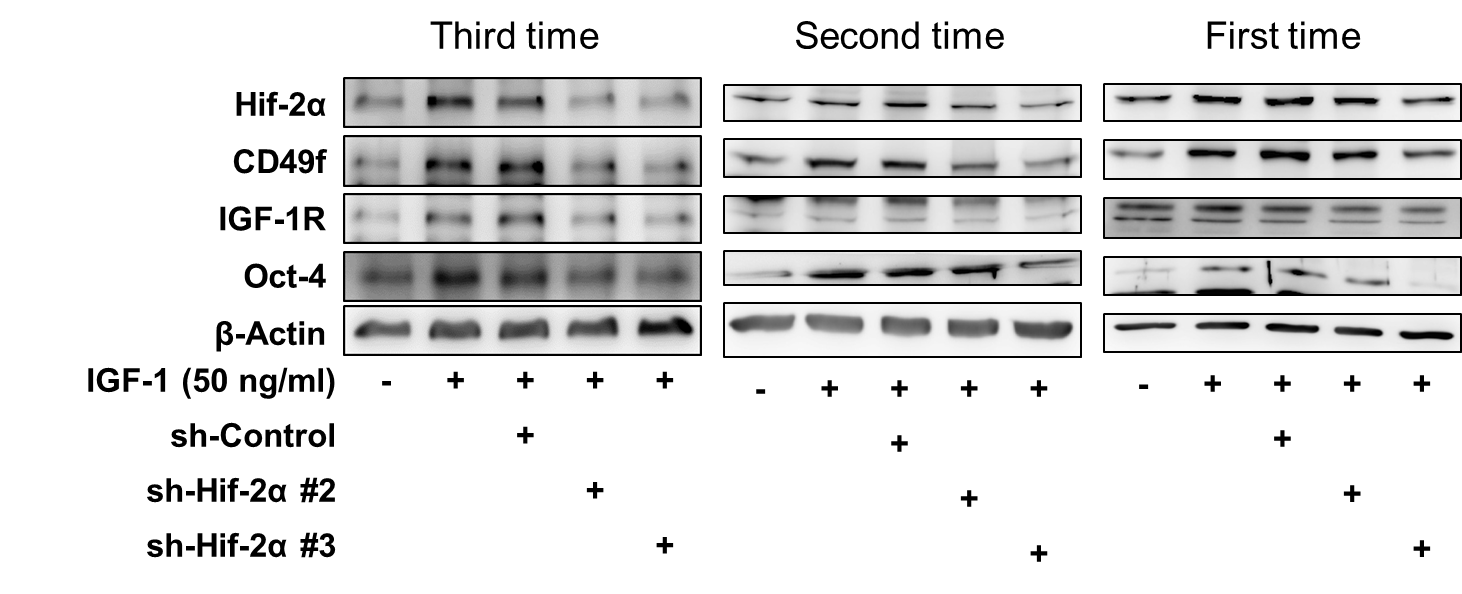
**

**Figure S8: The repeated three times data of Figure 2D.** Effect of shHif-2α on the expression of CD49f, IGF-1R, and Oct-4 in IGF-1-treated CD49f^+^AP^+^GSCs in the absence of laminin.


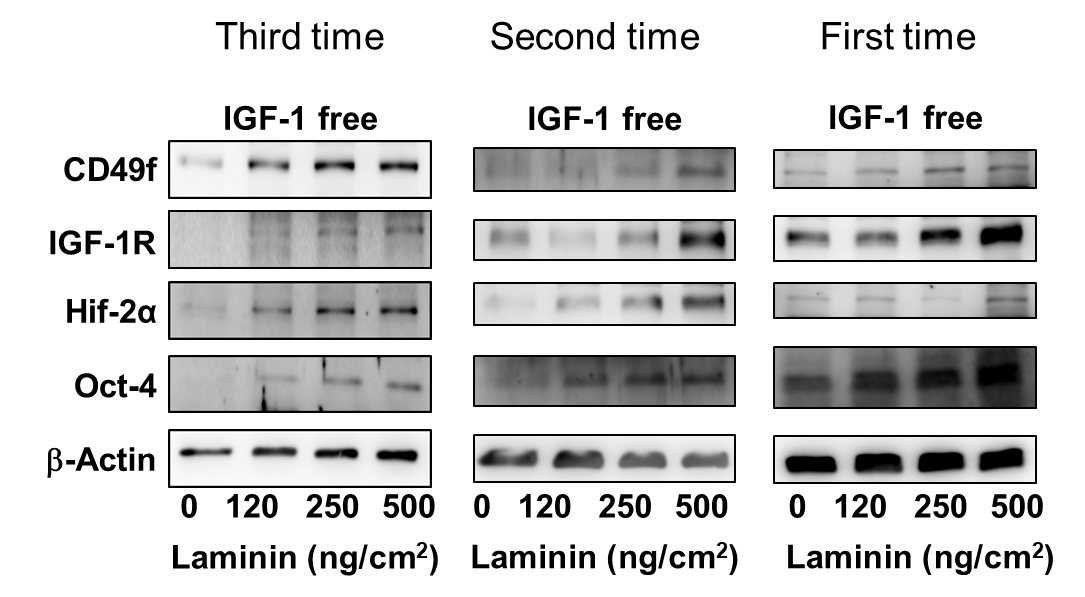


**Figure S9: The repeated three times data of Figure 3A.** Dose-dependent effects of laminin (0, 120, 250, and 500 ng/cm^2^) on the protein expression of CD49f, IGF-1R, Hif-2α, and Oct-4 in CD49f^+^AP^+^GSCs in the absence of IGF-1.


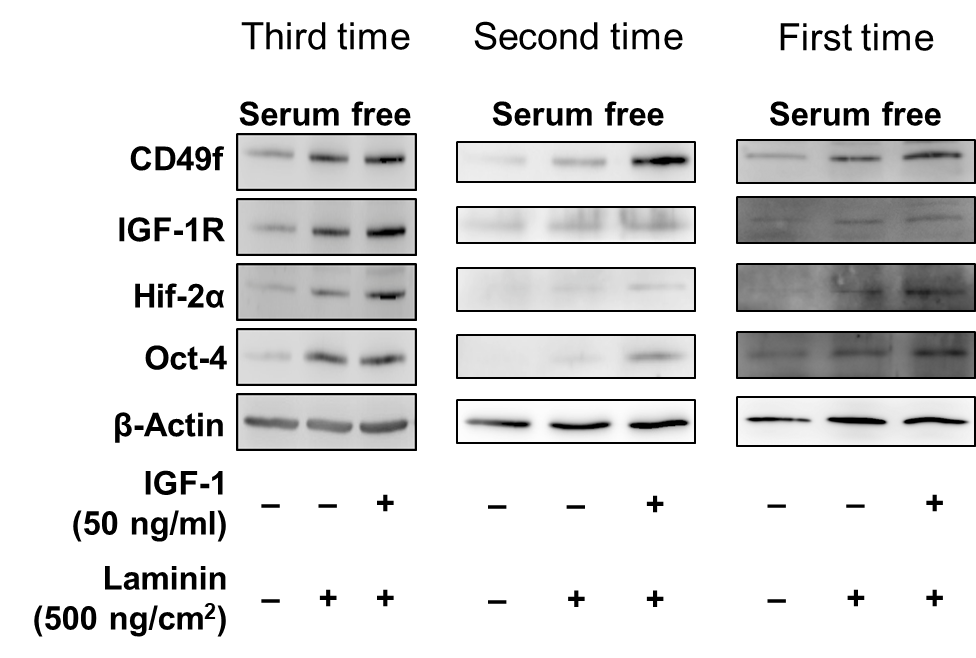


**Figure S10: The repeated three times data of Figure 3B.** Effects of laminin (500 ng/cm^2^) with or without IGF-1 (50 ng/mL) treatment on the expressions of IGF-1R, CD49f, Hif-2α, and Oct-4.

**
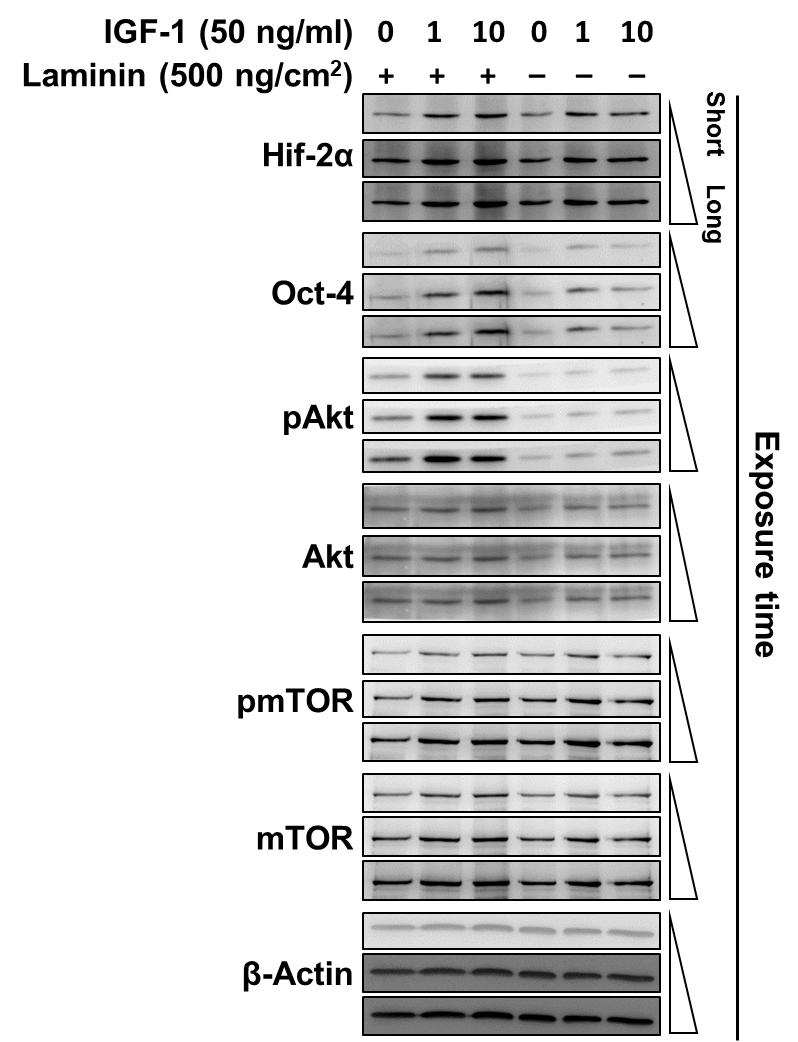

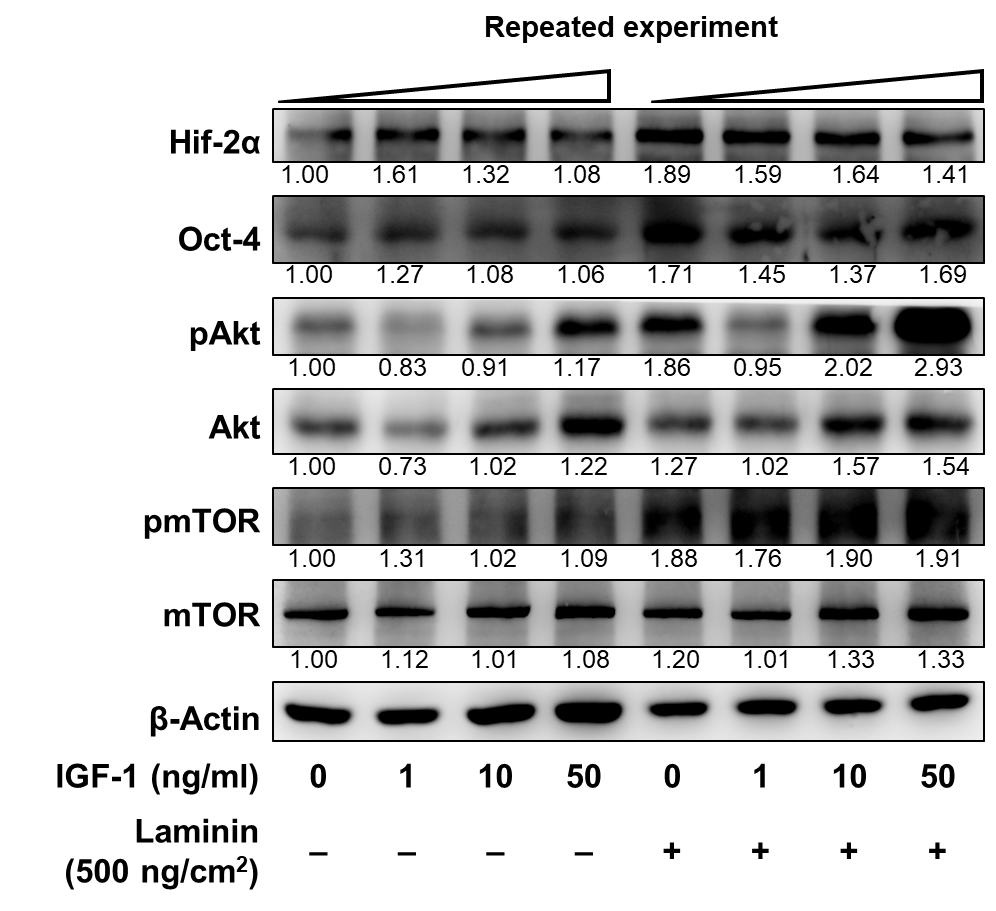
**

**A**

**B**

**Figure S11: The original western blot data of Figure 3D with different exposure times and repeated result. (A-B)** Dose-dependent effects of IGF-1 (0, 1, and 10 ng/mL) with or without laminin (500 ng/cm^2^) treatment on the protein expression of Hif-2α, Oct-4, and on the Akt-mTOR signaling.

**
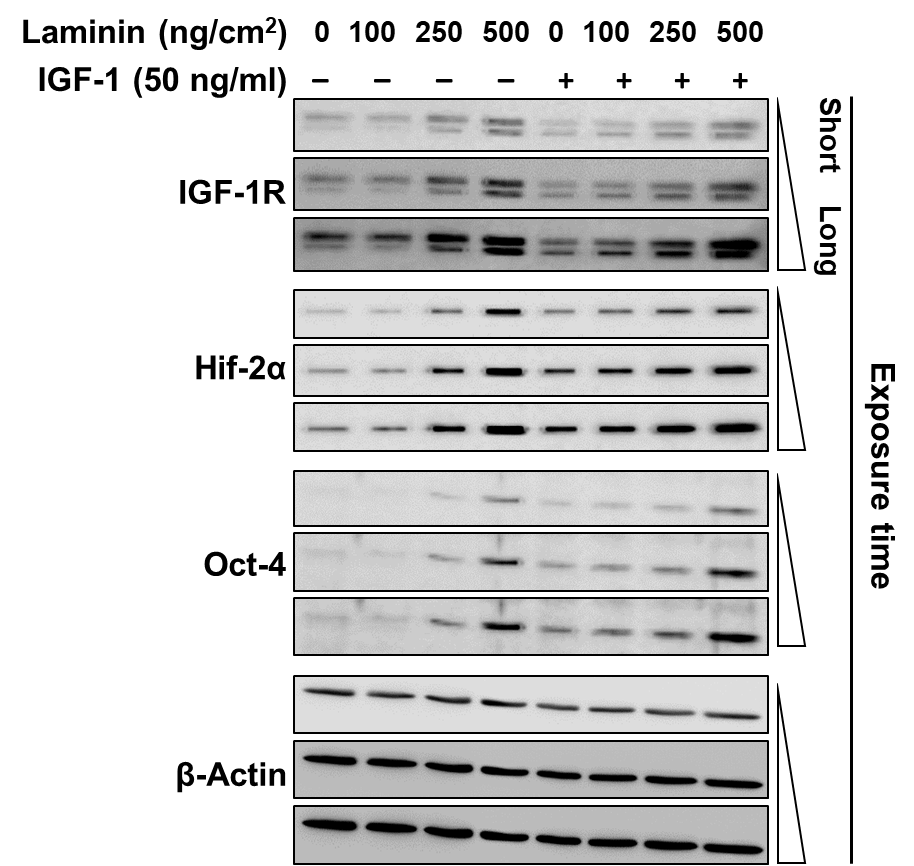
**

**Figure S12: The original western blot data of Figure 3E with different exposure times.** Dose-dependent effects of laminin (0, 100, 250, and 500 ng/cm^2^) with or without IGF-1 (50 ng/mL) treatment on the expression of IGF-1R, Hif-2α, and Oct-4.

**
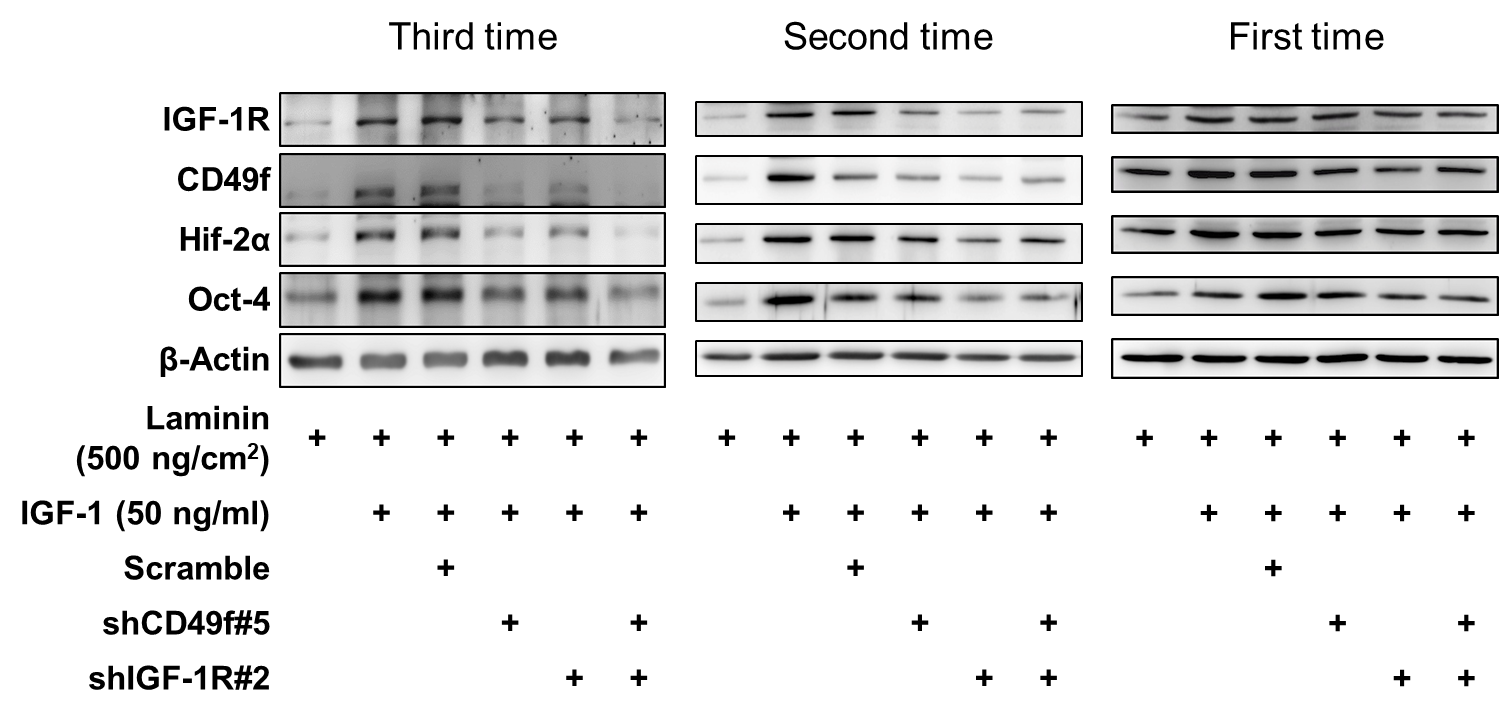
**

**Figure S13: The repeated three times data of Figure 4A.** Suppression effects of shCD49f and/or shIGF-1R on the expression of Hif-2α, Oct-4, CD49f, and IGF-1R in CD49f^+^AP^+^GSCs treated with IGF-1 (50 ng/mL) on laminin-coated substrates (500 ng/cm^2^).

**Supplemental Tables**

| **PCR primers and product size** | | | | | | |
| --- | --- | --- | --- | --- | --- | --- |
| **Gene** | **Accession** | **Forward Primers** | | **Reverse Primers** | **Annealing Temp (°C)** | **Product Size (bp)** |
| mOct-4 | NM_013633 | 5’-GGCGTTCTCTTTGGAAAGGTGTTC-3’ | | 5’-CTCGAACCACATCCTTCTCT-3’ | 62 | 313 |
| mNanog | NM_001289828 | 5’-AAAGGATGAAGTGCAAGCGGTGG-3’ | | 5’-CTGGCTTTGCCCTGACTTTAAG-3’ | 64 | 520 |
| mSox2 | NM_011443 | 5’-GAGTGGAAACTTTTGTCCGAGA-3’ | | 5’-GAAGCGTGTACTTATCCTTCTTCAT-3’ | 62 | 151 |
| mStella | NM_139218 | 5’-CAGCCGTACCTGTGGAGAACAAGAG-3’ | | 5’-AGCCCTGGGCCTCACAGCTT-3’ | 64 | 456 |
| mBlimp1 | [NM_007548](http://www.ncbi.nlm.nih.gov/entrez/viewer.fcgi?db=nucleotide&val=118131136) | 5’-GCCAACCAGGAACTTCTTGTGT-3’ | | 5’-AGGATAAACCACCCGAGGGT-3’ | 50 | 328 |
| mFragilis | NM_025378 | 5’-TTGCTCCGCACCATGAACCA-3’ | | 5’-TGAAGCACTCCAGGACCGGA-3’ | 58 | 461 |
| mPlzf | NM_001033324 | 5’-CCAGCCACACGGCTCTCAAGGCG-3’ | | 5’-ACTGGTATGGCGAGGCACCGTT-3’ | 60 | 312 |
| mMvh | NM_010029 | 5’-GCTCAAACAGGGTCTGGGAAG-3’ | | 5’-GGTTGATCAGTTCTCGAG-3’ | 56 | 269 |
| mGapdh | [G65758](http://www.ncbi.nlm.nih.gov/entrez/viewer.fcgi?db=nuccore&id=13624248) | 5’-ACCACAGTCCATGCCATCAC-3’ | | 5’-TCCACCACCCTGTTGCTGTA-3’ | 62 | 452 |
| **Real-time quantitative PCR primers and product size** | | | | | | |
| **Gene** | **Accession** | **Forward Primers** | **Reverse Primers** | | **Product Size (bp)** | |
| mOct-4 | NM_013633 | 5’-TGAAGTTGGAGAAGGTGGAACCAAC-3’ | 5’-CCAAGGTGATCCTCTTCTGCTTCAG-3’ | | 111 | |
| mCD49f | NM_001277970 | 5’-CGGTCTCCGGAGTCGCTAAG-3’ | 5’-AAGGTTGCTGTGCCGAGGTT-3’ | | 100 | |
| mHif-2α | NM_010137 | 5’-AGCTTCAGATTCATTTTCAGAGCA-3’ | 5’-CCTTCGGACACATAAGCTCCTG-3’ | | 54 | |
| mNanog | NM_001289828 | 5’-CCTGTGATTTGTGGGCCTG-3’ | 5’-GACAGTCTCCGTGTGAGGCAT-3’ | | 78 | |
| mSox2 | NM_011443 | 5’-GAGTGGAAACTTTTGTCCGAGA-3’ | 5’-GAAGCGTGTACTTATCCTTCTTCAT-3’ | | 151 | |
| mBlimp1 | NM_007548 | 5’-AGTGCAATGTCTGTGCCAAG-3’ | 5’-GTGGGCGAGCTGAGTAAAAC-3’ | | 125 | |
| mFragilis | NM_025378 | 5’-CCCAAACTACGAAAGAATCAAGG-3’ | 5’-GGGCTCCAGTCACATCACC-3’ | | 239 | |
| mPlzf | NM_001364543 | 5’-CTCCCCTGCTTACTCCTTTG-3’ | 5’-GACCTGCCTTCCTCTTCTTC-3’ | | 170 | |
| mMvh | NM_001145885 | 5’-CGCCAAACCCTTTTATTCAG-3’ | 5’-CCCAACAGCGACAAACAAG-3’ | | 96 | |
| mIgfals | NM_008340 | 5’-GTAGACGGCCCTTGCTCACT-3’ | 5’-TGAACTGCAAAAGACGCTGA-3’ | | 238 | |
| mLamc3 | NM_011836 | 5’-AAATGCTGCCTCTCTCTCCTC-3’ | 5’-CTCCTGCTTGTGTGCTTCTG-3’ | | 205 | |
| mMmp25 | NM_001033339 | 5’-ACTGGCGGTATGACGAGGT-3’ | 5’-GGTGCCCTTGAAAAAGTAAGTG-3’ | | 137 | |
| mFgf12 | NM_183064 | 5’-CCACAAATAAAAGCAGGACAAAG-3’ | 5’-TGGAGCAAAGACCAAGAAGC-3’ | | 126 | |
| mLama2 | NM_008481 | 5’-TCCAGCCAAACCATCAGTC-3’ | 5’-ATTCCTAACACAGCCATCCAG-3’ | | 138 | |
| mLamb1 | NM_008482 | 5’-GGATTTTGACCGTGATGTCC-3’ | 5’-CCTCGTTGCTCTTGTCCAC-3’ | | 161 | |
| mMmp27 | NM_001310717 | 5’-CAAATCCATCCACACACTCG-3’ | 5’-CATCATACCTCCAGCACCAG-3’ | | 119 | |
| mArhgef33 | NM_001145452 | 5’-CTTTCCCATTTCCAGATTTTAGG-3’ | 5’-TTTACCCTCCTCTTACCACTCG-3’ | | 195 | |
| mMmp9 | NM_013599 | 5’-CCTGGAACTCACACGACATC-3’ | 5’-CGGTTGAAGCAAAGAAGGAG-3’ | | 194 | |
| mβ-2M | NM_009735 | 5’-CCGAACATACTGAACTGC-3’ | 5’-AGAAAGACCAGTCCTTGC--3’ | | 185 | |

**Table S1. PCR primer sequence and product size**

**Table S2. List of antibodies**

| **Protein** | **Assay** | **Ab Cat. No.** | **Company** | **Origin** | **Dilution** | **Incubation Period** | **Protein Size (kDa)** |
| --- | --- | --- | --- | --- | --- | --- | --- |
| Oct-4 | ICC | sc-5279 | Santa Cruz Biotech | mouse | 1:100 | overnight, 4°C | 43 |
| CD49f | ICC | ab181551 | abcam | rabbit | 1:200 | overnight, 4°C | 127 |
| Oct-4 | IHC | sc-5279 | Santa Cruz Biotech | mouse | 1:100 | overnight, 4°C | 43 |
| Hif-2α | IHC | NB100-122 | Novus | rabbit | 1:100 | overnight, 4°C | 118 |
| CD49f | IHC | ab181551 | abcam | rabbit | 1:100 | overnight, 4°C | 127 |
| Laminin | IHC | ab11575 | abcam | rabbit | 1:100 | overnight, 4°C | 250 |
| Oct-4 | WB | sc-5279 | Santa Cruz Biotech | mouse | 1:1000 | overnight, 4°C | 43 |
| Hif-2α | WB | NB100-122 | Novus | rabbit | 1:1000 | overnight, 4°C | 118 |
| CD49f | WB | #3750 | Cell Signaling | rabbit | 1:1000 | overnight, 4°C | 125,150 |
| IGF-IRβ | WB | sc-713 | Santa Cruz Biotech | rabbit | 1:1000 | overnight, 4°C | 97 |
| pAKT | WB | #9271 | Cell Signaling | rabbit | 1:1000 | overnight, 4°C | 60 |
| AKT | WB | Sc-8312 | Santa Cruz | rabbit | 1:1000 | overnight, 4°C | 56 |
| pmTOR | WB | #2971 | Cell Signaling | rabbit | 1:1000 | overnight, 4°C | 289 |
| mTOR | WB | #2972 | Cell Signaling | rabbit | 1:1000 | overnight, 4°C | 89 |
| β-Actin | WB | A5441 | Sigma | mouse | 1:500 | overnight, 4°C | 42 |

**Supplemental Experimental Procedures:**

**Cultivation of mouse AP^+^GSCs in serum-free culture medium**

Newborn ICR mice (0 – 2day postpartum [dpp]) were obtained from the National Laboratory Animal Center and National Applied Research Laboratories (Taipei, Taiwan). Mouse AP^+^GSCs were generated as previously described . In brief, the testes of 0 – 2 dpp newborn ICR mice were collected and briefly washed in Hank’s buffer (Gibco BRL, Grand Island, NY, USA) containing penicillin (100 units/mL) and streptomycin (100 μg/mL) before treatment with 0.1% protease type-XIV (Sigma, St. Louis, MO, USA) in MCDB-201 medium (Sigma) at 4°C for 16 – 20 h. Digested tissues were transferred to Minimum Essential Medium Eagle (MEM), Spinner Modification (S-MEM) medium (Sigma) containing 10% fetal calf serum and filtered using a 70 μm nylon cell strainer to remove cell debris. In general, one testis yielded approximately 1.5 × 10^5^ cells. For AP^+^GSCs colony formation, all the testicular cells were resuspended in basic culture medium (serum-free) comprising MCDB-201 medium supplemented with 1× insulin, transferrin, and selenium as well as 10 ng/mL epidermal growth factor (EGF; Gibco BRL). Then, the testicular cells were seeded on a laminin (0 - 500 ng/cm^2^)-coated culture plate at a density of 8 × 10^4^ cells/cm^2^ and were incubated at 37°C for 7 days. AP^+^GSC colonies were collected for gene and protein expression analysis.

**Purification of CD49f^+^AP^+^GSCs from mouse neonatal testes**

The CD49f^+^AP^+^GSCs were purified using a magnetic-activated cell sorting system (MACS, Miltenyi Biotec, Bergisch Gladbach, Germany). Briefly, all testicular cells were resuspended in 0.5 mL of blocking buffer and then incubated with specific Phycoerythrin (PE)-conjugated primary antibodies against CD49f (10 mg/mL) for 1 h on ice. CD49f-labeled cells were washed twice with phosphate-buffered saline (PBS) containing 2% bovine serum albumin (BSA), and then incubated with anti-PE microbeads (Miltenyi Biotec) for 40 min at 4°C. Stained cells were separated using a MACS column (Miltenyi Biotec) according to the manufacturer’s recommended protocol. The CD49f-positive cells were then cultivated in serum-free medium and used in subsequent experiments. For hypoxia experiments, CD49f^+^AP^+^GSCs were cultivated in a 5% CO_2_ incubator under 5% (hypoxia) or 21% oxygen (normoxia) for 48h. For experiments with CD49f^+^AP^+^GSCs, the cells were first cultivated in serum-free, growth factors–free, and laminin-free medium, followed by the treatment of growth factors (such as IGF-1, EGF, or TGF-β) or inhibitors of interest.

**AP activity assay**

Clustered GSC colonies or MACS-purified CD49f^+^GSCs in serum-free medium were fixed with 3.7% paraformaldehyde for 2 min at room temperature. The AP activity of these GSCs was examined using an AP detection kit according to the manufacturer’s instructions (Chemicon, Hampshire, UK).

**Immunostaining**

To detect Oct-4 expression in the primary cultured cells, the cells were fixed in methanol: acetone (1:1) at room temperature for 10 min. For other antigens, cells or tissues were fixed with 3.7% paraformaldehyde at room temperature for 30 min. After fixation, the cells were rinsed twice with PBS, treated with PBS containing 0.1% Triton-X 100 (PBST) at room temperature for 10 min, and blocked using BSA (5 mg/mL) in PBST for 1 h at room temperature. For confocal spectroscopic fluorescence, the AP^+^GSCs were immunoprobed at 4°C overnight by using the following antibodies: anti-Oct-4 (sc-5279, from Santa Cruz Biotechnology, Santa Cruz, CA, USA), anti-CD49f (ab181551, from Abcam, and anti-β-Actin (A544, Sigma). The experimental conditions are listed in Supplementary Table S2. Specific labeling of primary antibodies was performed using Cy3- or FITC-conjugated secondary antibodies (Jackson ImmunoResearch, West Grove, PA, USA). The nuclei were counterstained using 4′,6-diamidino-2-phenylindole (Sigma). All the cells were covered with an antifading reagent (Vector Laboratories, Burlingame, CA, USA) and analyzed using a fluorescence microscope (Olympus, Melville, NY, USA).

**Effect of IGF-1R signaling inhibitors on CD49f expression**

The inhibitors of IGF-1R signaling that were used in this study were cyclolignan picropodophyllin (PPP, 1 μM, CalBiochem, San Diego, CA, USA), an IGF-1R phosphorylation inhibitor; LY294002 (10 μM, Cell Signaling, Danvers, MA, USA), a PI3 kinase inhibitor; and rapamycin (50 nM, Sigma-Aldrich, St. Louis, USA), an mTOR inhibitor. For examination of CD49f expression, particularly in the AP^+^GSC cells, CD49f^+^AP^+^GSCs were collected using MACS and cultured in the absence or presence of laminin (0–500 ng/cm^2^, Sigma-Aldrich) and IGF-1 (50 ng/mL, Peprotech, Rocky Hill, NJ, USA) for 24 h. Cell lysates were collected for sodium dodecyl sulfate–polyacrylamide gel electrophoresis (SDS-PAGE) and Western blotting analysis.
